# Molecular Aharonov–Bohm-type interferometers based on porphyrin nanorings†

**DOI:** 10.1039/d4sc07992b

**Published:** 2025-01-29

**Authors:** Chi Y. Cheng, Gil Harari, Igor Rončević, Juan E. Peralta, Harry L. Anderson, Andrew M. Wibowo-Teale, Oded Hod

**Affiliations:** a School of Chemistry, University of Nottingham, University Park Nottingham NG7 2RD UK; b School of Chemistry, The Sackler Centre for Computational Molecular and Materials Science, Tel Aviv University Tel Aviv 6997801 Israel; c Department of Chemistry, University of Oxford, Chemistry Research Laboratory Oxford OX1 3TA UK; d Department of Physics, Central Michigan University Mount Pleasant MI 48859 USA

## Abstract

A goal of molecular electronics and spintronics is to create molecular devices that change their conductance in response to external stimuli. The Aharonov–Bohm (AB) effect implies that an electronic device formed from a quantum ring and metallic leads will exhibit such behavior under external magnetic fields. At first sight, it appears that unrealistically large fields would be required to significantly alter the conductance of a molecular ring. However, the sensitivity of a molecular AB interferometer to magnetic fields can be increased by weakening the coupling between the molecular ring and the metallic leads. An ideal molecular ring for an AB interferometer has a large radius (to encompass a larger fraction of the AB flux quantum), and a small effective mass (high electron mobility) to enhance its response to magnetic fields. Here, we use computational modelling to demonstrate that recently synthesized zinc porphyrin nanorings, with radii of 2–9 nm, could behave as molecular AB interferometers at achievable magnetic field strengths (5–10 T), if weak ring-lead coupling is used. Building on our recently developed semi-empirical approach, which incorporates the effects of finite magnetic fields on the electronic structure, we develop a transport computational platform that allows us to identify sharp Fano resonances in the transmittance probability of porphyrin nanorings that could be exploited to control the current with an applied magnetic field. These resonances are rationalized in terms of a magnetic field-induced delocalization of the molecular orbitals. Our findings indicate that molecular AB interferometry should be feasible with current experimental capabilities.

## Abstract 2

A goal of molecular electronics and spintronics is to create molecular devices that change their conductance in response to external stimuli.

## Graphical abstract

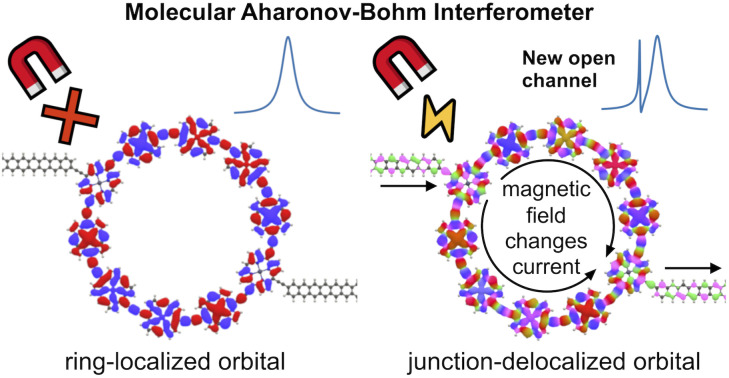

## 1 Introduction

**Fig. 1:**
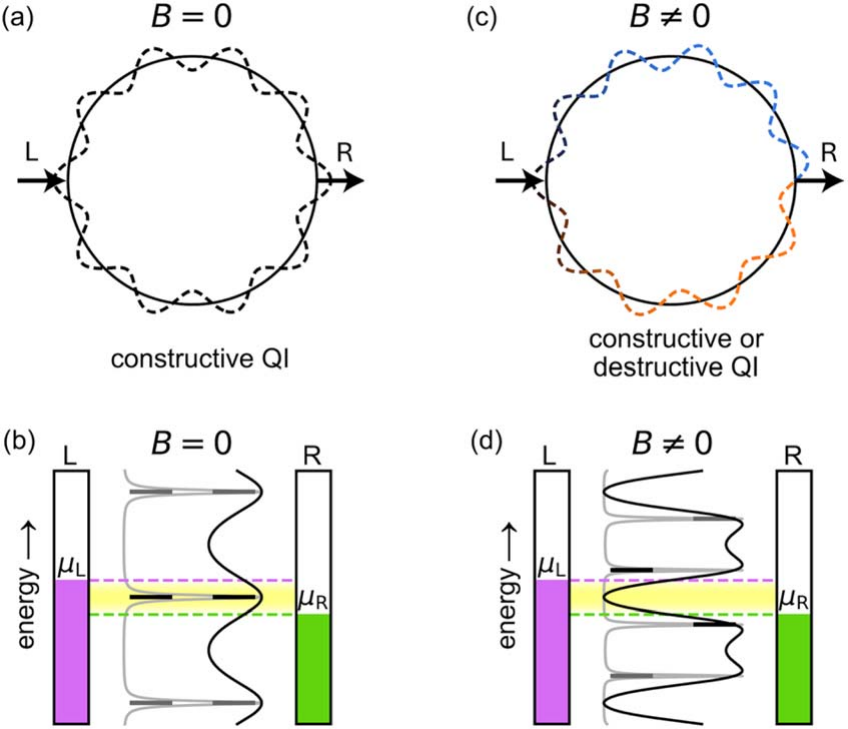
Principle of a symmetric AB interferometer. In the absence of a magnetic field (a and b), the two paths from the left (L) to the right (R) lead along a molecular ring are equivalent (a). This results in constructive QI and high conductance (b) if a conducting state (black lines) resides within the Fermi transport window (yellow shaded region) between the chemical potentials of the left (*μ*_L_, purple) and right (*μ*_R_, green) leads. A magnetic field, *B* ≠ 0, perpendicular to the ring (c and d), induces a phase difference between the two paths (c) and a splitting of the degenerate energy levels of the ring (d), which may result in destructive QI. In (b) and (d), transmittance functions in the weak and strong ring-lead coupling regimes are shown in gray and black, respectively. Zero electronic temperature is assumed for simplicity.

Aharonov and Bohm predicted that the phase of the wavefunction of electrons traversing a metal ring will be affected by the flux of an externally applied magnetic field threading through the ring, even if there is no magnetic field in the vicinity of the electrons.^1^ This theoretical result, which seemed paradoxical at the time, was fully supported by subsequent experimental and theoretical investigations, demonstrating that the vector potential (rather than the magnetic field) is a fundamental quantity in quantum mechanics.^2,3^ The term ‘Aharonov–Bohm (AB) effect’, however, is often used more generally to describe the fact that the ring current (or persistent current) flowing round a quantum ring oscillates as a function of the magnetic flux passing through the ring, without necessarily implying that the magnetic field is zero in the vicinity of the ring.^2–5^ An AB interferometer (Fig. 1) consists of a quantum ring attached to external leads, such that a current can be driven through it and controlled by the application of a magnetic field perpendicular to the ring.^4,5^ This magnetic control has been used to measure the quantum coherent properties of electrons,^5^ and it is predicted to enable other unique functionalities, including spin filtering in two-terminal setups,^6^ and current and spin routing in three-terminal setups.^7^

In the absence of a magnetic field, the upper and lower paths of a quantum ring from the left (L) to the right (R) lead, through a symmetrically connected AB interferometer, are equivalent (Fig. 1a), resulting in constructive quantum interference (QI) and high conductance (Fig. 1b). A magnetic field *B* perpendicular to the ring plane breaks the symmetry, as the electron accumulates an opposite phase when traversing the ring in a clockwise or counterclockwise path (Fig. 1c). This results in periodically alternating constructive or destructive QI and consequently high or low conductance (Fig. 1d), depending on the magnetic flux *Φ*, which is given by the product of the perpendicular magnetic field, *B*, and the cross-sectional area of the ring of radius *r*,

1 *Φ*(*B*) = *B*π*r*^2^.

The flux required for a full AB cycle between constructive and destructive interferences is the magnetic flux quantum:^8^

2 *Φ*_0_ = 2πℏ / *e* ≈ 4.1 × 10^5^ T Å^2^,

where *e* is the electron charge, and ℏ is the reduced Planck constant. As molecular rings usually have radii on the order of 1–10 Å, building molecular AB interferometers was commonly thought to be beyond reach, as extremely large magnetic fields (∼10^4^–10^5^ T) would be required to reach the flux quantum. As a result, most AB interferometers investigated over the last few decades have been mesoscopic.^9^ Nonetheless, optical signatures of the AB effect have been observed in systems of nanoscale cross section, such as single-walled carbon nanotubes.^10–12^

If the ring is weakly coupled to external metallic leads, the presence of a magnetic field may significantly alter the molecular conductance, even when the magnetic flux through the ring is considerably smaller than a full flux quantum.^6,7,13–15^ A simple explanation for this effect can be given using the particle-in-a-ring (PIR) model.^16,17^ The electronic spectrum of an isolated PIR consists of a series of doubly degenerate eigenstates and a non-degenerate ground state (*n* = 0), with angular momentum quantum numbers ±*n* and energies,3
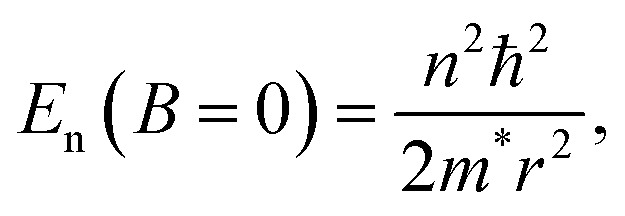
where m* is the effective particle mass. By coupling the ring to biased external leads, electronic transport will occur through broadened ring eigenstates of Lorentzian shape, with widths that increase with the strength of the ring-lead couplings. If a conducting broadened state, whose orbital spans the entire junction, resides within the Fermi transport window (defined as the region between the chemical potentials of the left (*μ*_L_) and right (*μ*_R_) leads; Fig. 1b), then the system will have high conductance. When an external magnetic field is applied across the ring, the (±*n*) degenerate energy levels split according to4
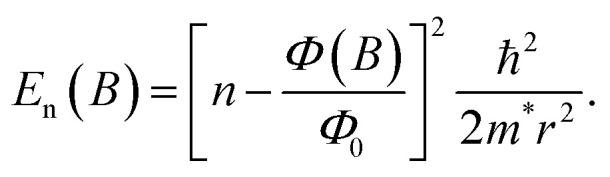
For a sufficiently large flux *Φ*/*Φ*_0_, the states will exit the Fermi transport window and conduction will drop (Fig. 1d). Further increases in the field intensity push adjacent states back into the Fermi window, again increasing the conductance until a full AB cycle is obtained.

For sufficiently strong ring-lead couplings, the ring state widths become comparable to the width of the Fermi window and conductance variations are only apparent over the entire AB period (black curves in Fig. 1b and d). For weaker ring-lead couplings, conductance variations will occur when the relatively narrow eigenstates approach the edges of the Fermi transport window (gray curves in Fig. 1b and d). As a result, the conductance becomes sensitive to variations in the magnetic field that are much smaller than the flux quantum. This understanding opens the door for achieving AB interferometry in molecular rings and utilizing it for the miniaturization of electronic switching and routing devices. Nonetheless, previous literature considered mainly simplistic model systems of weakly coupled molecular rings, and focused on the phenomenological investigation of the effect, rather than on its practical utilization.^6,7,13–15,18,19^

To take advantage of the AB effect, one needs to maximize the splitting of the energy eigenvalues, *E*_n_, with the magnetic field. From eqn (1)–(4), this splitting is given by:5
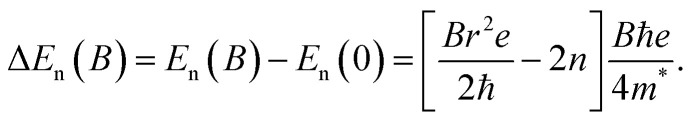
Therefore, the PIR model implies that good candidates for molecular AB interferometers should have large ring radii to enable access to a larger portion of the full AB cycle (see eqn (1)), and a small effective mass (high electron mobility, see eqn (5)) to enhance the response to the applied magnetic field.

**Fig. 2:**
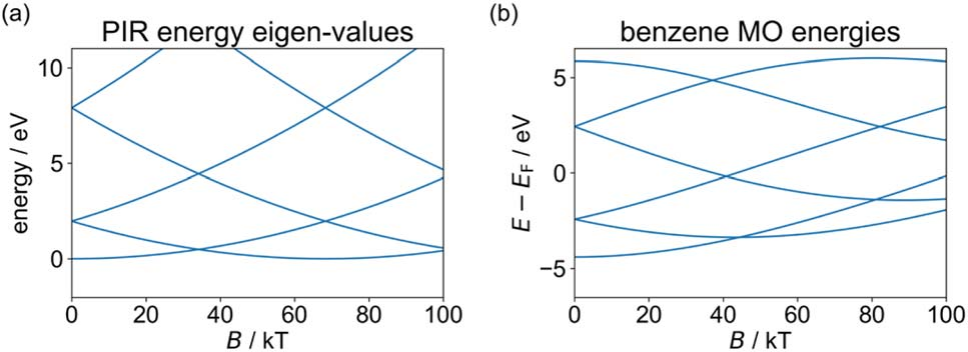
Magnetic field dependence of the energy eigenvalues of (a) a particle in a ring (calculated *via*eqn (4) with an effective ring radius of 1.39 Å – the radius of the circumcircle of the benzene hexagon used in the GFN1-xTB-M1 calculations, and effective masses of m* = *m*_e_ for all states), and (b) the benzene molecule (calculated using the GFN1-xTB-M1 method and plotted relative to the center of the HOMO–LUMO gap in the absence of a magnetic field). For comparison purposes, in the molecular calculations the same benzene structure (obtained by geometry optimization in the absence of a magnetic field) is used throughout, and only benzene energy eigenvalues of MOs with contributions from the p_*z*_ atomic orbitals are shown while spin-Zeeman effects are removed (results including spin-Zeeman interactions appear in ESI Section S.1†).

The degenerate orbitals of a conjugated ring system, such as benzene, split under an external field in a way reminiscent of the PIR model, due to their opposite orbital angular momentum. This is illustrated in Fig. 2, where we compare the effect of the magnetic field (*B*) on the PIR energy eigenvalues obtained *via*eqn (4) and on the orbital energies of benzene, calculated using our tight-binding semiempirical GFN1-xTB-M1 method (discussed below in more detail).^20^ The qualitative agreement between the phenomenological PIR model and the results calculated for the molecular ring allows us to introduce the concept of an effective mass for the π-electrons in the molecular ring, as the mass that provides good agreement between the PIR model and the molecular energy bands. This simple example demonstrates that realistic molecules can present PIR-like behavior. Nevertheless, achieving a full AB cycle in benzene would require an unrealistically large field of ∼80 kT. Clearly, even by reducing the coupling to the leads, the sensitivity of any benzene-based AB interferometric device would be insufficient to respond to a field of around 10 T, and larger molecules are needed.

**Fig. 3:**
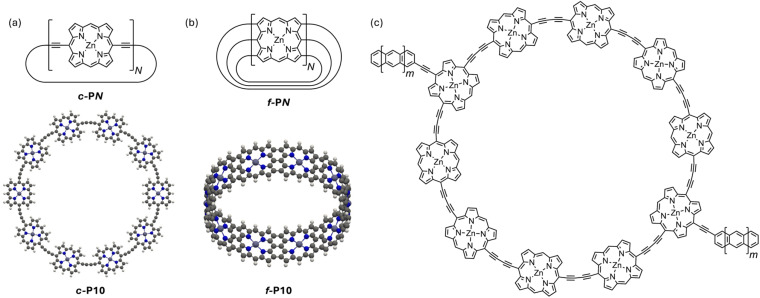
Chemical structures of *c*-P*N* (a, top) and *f*-P*N* (b, top) nanorings and optimized field-free geometries of (a, bottom) *c*-P10 (planar geometry) and (b, bottom) *f*-P10 molecular rings, calculated at the GFN1-xTB-M1 level of theory. (c) A section of the AB interferometer constructed from the *c*-P10 molecular ring with oligoacene leads. When these structures are studied experimentally, they have bulky substituents at the *meso* positions to confer solubility and prevent aggregation; these solubilizing groups do not affect the electronic structure in the energy range relevant to transport, so they are not included in this study.

Butadiyne-linked cyclic porphyrin oligomers (*c-*P*N*, where *N* is the number of porphyrin units, Fig. 3a) are promising molecular nanorings for use in AB interferometers because they have been prepared in a variety of different sizes with radii of up to 104 Å (50 porphyrin units)^21^ and shown to feature extensive electronic delocalization.^22^ Rings of up to 12 porphyrin units (*c-*P12, *r* ≈ 25 Å) were shown experimentally to exhibit global aromatic and anti-aromatic ring currents depending on their oxidation state.^23–25^ This indicates that their electronic wavefunction is coherently delocalized around the entire ring, with an electronic structure analogous to that of a PIR. In larger rings, structural symmetry breaking, similar to the Peierls distortion, may lead to localization of the molecular orbitals and an increase of the effective electronic mass. However, scanning tunnelling spectroscopy experiments on a butadiyne-linked 40-porphyrin nanoring (*c-*P40, *r* ≈ 83 Å) on a silver surface, indicate that its electronic states remain delocalized around the entire ring.^24^

Edge-fused porphyrin nanobelts (*f*-P*N*, Fig. 3b) constitute another molecular ring family, which is predicted to exhibit lower effective electronic masses^26^ and greater electronic delocalization.^27^ These properties make them even more interesting in the context of molecular AB interferometry. While *f*-P*N* nanobelts have not yet been synthesized, the corresponding linear ribbons have been studied extensively^28–31^ and are known to mediate coherent ballistic charge transport.^30,31^

Motivated by the potential applicability of porphyrin nanoring-based AB interferometers, in the present study we explore the conductance properties of realistic molecular rings and possible routes to control their response with experimentally accessible magnetic fields (5–10 T), focusing on macrocycles constructed from zinc porphyrin units. The closed-shell d^10^ zinc ions do not have a strong influence on the molecular electronic structure, and nanorings containing other closed-shell metal cations are expected to display qualitatively similar behavior.

## 2 Computational methods

We have developed a nonequilibrium Green's function (NEGF)^8,32–34^ approach for calculating the electronic current flowing through molecular junctions under finite bias and magnetic field. We implemented this approach within the QUEST code^35^ that provides a multitude of different electronic structure methods, including current density-functional theory (CDFT),^36,37^ Hartree–Fock,^38,39^ and extended tight-binding methods (*e.g.* GFN1-xTB)^40–42^ with a non-perturbative treatment of the external magnetic field (GFN1-xTB-M1).

When modelling ring currents in *c-*P*N* porphyrin nanorings using DFT, it is crucial to include the correct proportion of exact exchange (EE), and this has led to debate about the best choice of density functionals.^43–45^ Hybrid density functionals with low levels of EE predict too much delocalization, underestimating band gaps and overestimating the magnitude of ring currents.^27^ As the GFN1-xTB method (and its M1 variant) produce results similar to pure density functionals (0% EE),^40^ we expect to see an overestimation of AB effect, just as GFN1-xTB underestimates effective masses for infinite linear porphyrin ribbons when compared to hybrid DFT calculations.^46^

To verify that our GFN1-xTB-M1 approach provides results that are reasonably consistent with hybrid CDFT for the systems considered, we calculated the magnetic field dependence of the orbital energies of the small *c-*P4 and *f-*P4 nanorings with both tight-binding and CDFT (PBE0, 25% EE) approaches. The results, presented in ESI Section S.2,† demonstrate good agreement between these two levels of theory. This is promising, as 25% EE was found to be suitable for modelling *f*-PN systems,^27^ while for neutral *c*-P*N* rings both GFN1-xTB and PBE0 predicted a Peierls-type distortion, which is essential for an accurate account of the electronic structure.^43–45^ Encouraged by these results, and taking into account the high computational cost of applying CDFT to these large systems, we used the NEGF transport approach in conjunction with the tight-binding GFN1-xTB-M1 method under external magnetic fields, a method that we term NEGF+GFN1-xTB-M1.

To perform the transport calculations, the molecular junction is divided into three sections: the left lead (L, source), the right lead (R, drain), and the extended molecule (M), which consists of the molecular ring and adjacent lead sections chosen to be sufficiently large to buffer the leads from the ring. The total current, *I*, flowing across the molecule can be evaluated using the Landauer formalism, according to which the current is proportional to the transmittance probability of an electron to traverse the system at some energy, *E*:^8,32–34^6
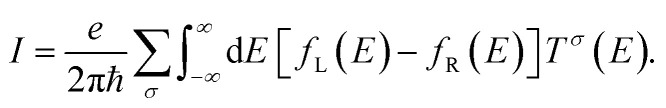
Here, *f*_L/R_(*E*) is the Fermi–Dirac distribution associated with the left or right reservoirs, given by:

7 *f*_L/R_(*E*) = [1 + e^(*E*−*μ*_L/R_)/(*k*_B_*T*_L/R_)^]^−1^,

where *μ*_L/R_ is the chemical potential and *T*_L/R_ is the electronic temperature of the left or right reservoir, respectively; [*f*_L_(*E*) − *f*_R_(*E*)] is the Fermi transport window, and *T*^*σ*^(*E*) is the transmittance probability of spin channel *σ* = {*α*,*β*}. The transmittance probability through the molecular junction can be evaluated *via*:

8 *T*^*σ*^(*E*) = *Tr*[**Γ**_L_^*σ*^(*E*)**G**_M_^*r*,*σ*^(*E*)Γ_R_^*σ*^(*E*)**G**_M_^*a*,*σ*^(*E*)],

where **Γ**_L/R_^*σ*^(*E*) are the left or right lead broadening matrices, and **G**_M_^*r*,*σ*^(*E*) = [**G**_M_^*a*,*σ*^(*E*)]^†^ are the extended molecule retarded and advanced Green's functions (see ESI Section S.3† for explicit expressions of the matrix representations of these operators). Importantly, under a finite magnetic field, a modified iterative procedure is required to generate the surface Green's functions of the leads, in order to account for non-translationally invariant matrix elements that arise when using London atomic orbitals (see ESI Section S.4† for further details). A schematic illustration of the different contributions to the integrand in eqn (6) is shown in Fig. 1b and d, in which the Fermi transport window is represented by the yellow shaded area between the horizontal dashed purple and green lines and the transmittance probability (for two example scenarios) as the black and gray curves.

We use a non-self-consistent NEGF approach, which neglects the effects of the bias potential on the electronic structure of the device. This approximation is found to be adequate for our purposes, since we consider relatively low bias voltages and weakly coupled molecular bridges (see ESI Section S.5†). Similar non-self-consistent approaches have been successfully implemented both in the Amsterdam Density Functional (ADF)^47–49^ and the DFTB+^50–52^ quantum chemistry package for transport calculations in the absence of a magnetic field.

## 3 Results and discussion

### 3.1 Response of molecular orbital energies to a magnetic field

We first examine the effect of an external magnetic field on the energy levels of isolated molecular rings. We consider both the planar butadiyne-linked porphyrin nanorings *c-*P*N* (results for non-planar geometries are similar, see ESI Section S.6†), and the edge-fused porphyrin nanobelts *f-*P*N*. Using the tight-binding GFN1-xTB-M1 method, we optimized planar *c-*P*N* and cylindrical *f-*P*N* molecules with 10, 20, 30, and 40 porphyrin monomer units in the absence of a magnetic field (see Fig. 3a and b for optimized *c-*P10 and *f-*P10 molecular ring structures), yielding nanorings with radii of 21.1, 42.8, 64.4, and 86.0 Å for the *c-*P*N* series and 13.3, 26.7, 40.0, and 53.3 Å for the *f-*P*N* series, respectively. The magnetic field strengths corresponding to one flux quantum for these radii are listed in Table 1 (*B*_0_, calculated from eqn (1) and (2)).

**Table 1:** Calculated radii, effective masses m* (for selected orbitals) and magnetic fields corresponding to one flux quantum *B*_0_ for several molecular nanorings

Nanoring	*r*/nm	m*/*m*_e_ (HOMO)	m*/*m*_e_ (LUMO)	*B*_0_/T
*c-*P10	21.1	−0.1081	0.1055	296
*c-*P20	42.8	−0.1083	0.1057	72
*c-*P40	86.0	−0.1084	0.1057	18
*f-*P10	13.3	−0.0134	0.0129	744
*f-*P20	26.7	−0.0073	0.0073	185
*f-*P40	53.3	−0.0059	0.0058	46

As the frontier orbital energies of the *c-*P*N* and *f-*P*N* nanorings respond to an external magnetic field similarly to their corresponding PIR eigenstates, we can fit their orbital energy functions around *B* = 0 to a quadratic function and determine the effective mass *via*eqn (4). For the LUMOs, we obtain effective masses of ∼0.1*m*_e_ for *c-*P*N* nanorings and ∼0.01*m*_e_ for *f-*P*N* nanobelts (see Table 1).^46^ Equivalent calculations for the HOMO yield negative effective masses, in analogy with hole quasiparticles^53^ in solid-state physics. The smaller m* obtained for the *f-*P*N* nanobelts is expected, given the larger coupling between its porphyrin monomer units. Unlike the *c-*P*N* series, which shows similar effective masses for all nanoring sizes considered, the *f-*P*N* series shows decreasing effective mass with increasing ring size. This is attributed to the fact that orbital overlap between neighboring porphyrin units is large and increases as the rings become larger and less strained. A similar method was used previously to estimate the effective masses of charge carriers in benzene (*m*_HOMO_ = 1.5*m*_e_) and in a C_576_ toroid (*m*_HOMO_ = 0.3*m*_e_).^54–56^ The lower values for *c-*P*N* and *f-*P*N* imply a greater sensitivity to a perpendicular magnetic field.

**Fig. 4:**
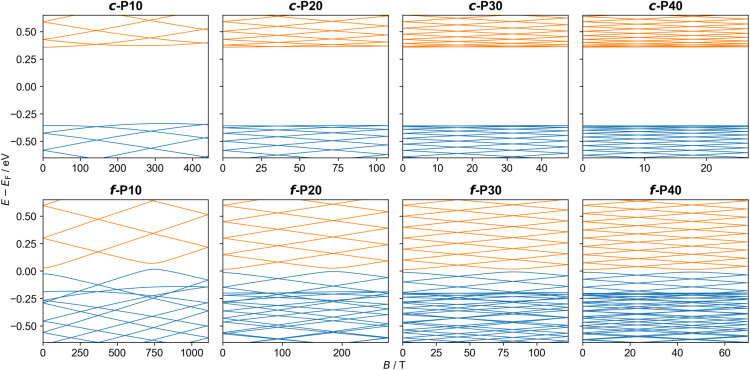
The α-spin orbital energies of planar *c-*P10, *c-*P20, *c-*P30, *c-*P40 (upper panels) and *f-*P10, *f-*P20, *f-*P30, *f-*P40 (lower panels) molecular rings as a function of the perpendicular magnetic field strength. Energies are plotted relative to the corresponding field-free HOMO–LUMO gap center. Occupied and unoccupied orbitals are shown in blue and orange, respectively. Results for the β-spin orbital energies show minor differences due to the spin-Zeeman interaction (see ESI Section S.7†).

Under an experimentally accessible field strength of 10 T, a splitting of the low-lying energy eigenvalues of up to ∼20 meV is predicted (see Table 2). Due to their lower charge carrier effective mass, the orbitals of the *f-*P*N* rings present a stronger magnetic response than their *c-*P*N* counterparts (see Fig. 4). However, the *c-*P*N* rings have larger radii (at a given value of *N*), which somewhat counteract their larger effective mass. The results of these two opposing effects can be observed in Table 2, where below *N* ≈ 20 the butadiyne-linked *c-*P*N* rings exhibit a larger response than the corresponding *f-*P*N* rings under a perpendicular magnetic field of 10 T. These results demonstrate that both the chemical nature and the cross-sectional area of molecular rings can be tailored to control their response to externally applied magnetic fields.

**Table 2:** Changes in the α-spin orbital energies, Δ*E*/meV, (with respect to the field-free case) of planar *c-*P10, *c-*P20, c-P30, *c-*P40 and *f-*P10, *f-*P20, *f-*P30, *f-*P40 nanorings due to a perpendicular magnetic field of 10 T. Results for the β-spin orbital energy changes show minor differences due to the spin-Zeeman interaction (see ESI Section S.7)

Orbital	*c-*P10	*c-*P20	c-P30	*c-*P40^a^	*f-*P10	*f-*P20	*f-*P30	*f-*P40
LUMO+4	7.0	9.9	11.2	12.2	4.6	8.7	12.7	16.7
LUMO+3	−5.8	−8.3	−8.7	−8.5	−3.4	−7.5	−11.5	−15.5
LUMO+2	5.2	6.1	6.7	7.4	4.6	8.6	12.6	16.5
LUMO+1	−3.9	−4.3	−4.0	−3.3	−3.4	−7.4	−11.4	−15.3
LUMO	0.7	1.0	1.4	2.1	0.9	2.6	5.4	8.6
HOMO	0.5	0.2	−0.3	−0.9	0.3	−1.4	−4.2	−7.5
HOMO−1	4.9	5.3	5.0	4.4	0.6	8.4	12.4	16.2
HOMO−2	−3.8	−4.8	−5.4	−6.1	2.6	−7.2	−11.2	−15.1
HOMO−3	6.7	9.2	9.7	9.4	−1.4	0.5	12.3	16.3
HOMO−4	−5.5	−8.4	−9.8	−10.8	4.4	2.7	−11.1^a^	−15.1

a For all *c-*P40 orbitals and the *f-*P30 HOMO−4, the 10 T magnetic field was sufficiently large to induce state crossings. In these cases, we calculate the energy changes by following the orbital through the crossing.

### 3.2 Molecular AB interferometry

Having explored the magnetic response of isolated molecular rings, we now evaluate their performance as AB interferometers. We focus on the *c-*P10 molecular ring, which is experimentally well characterized.^23,57–59^ The neutral *c-*P10 nanoring has an electronic structure similar to that of an anti-aromatic compound, with a circuit of 4*k* (*k* = 30) π-electrons, non-degenerate HOMO and LUMO, and doubly degenerate orbital pairs below and above (assuming high structural symmetry). Experimental NMR spectroscopy has shown that *c-*P10 is non-aromatic in its neutral state, globally aromatic in its 6+ oxidation state and globally anti-aromatic in its 8+ oxidation state, following the Hückel rule.^23^ In the context of molecular interferometry, an important advantage of *c-*P10 is its ability to assume a planar conformation on surfaces, making it suitable for junction fabrication.^57^ Furthermore, chemical routes have already been developed for functionalizing its diagonally opposite porphyrin units, which could be extended to add anchoring groups for electrode connection.^58–60^

**Fig. 5:**
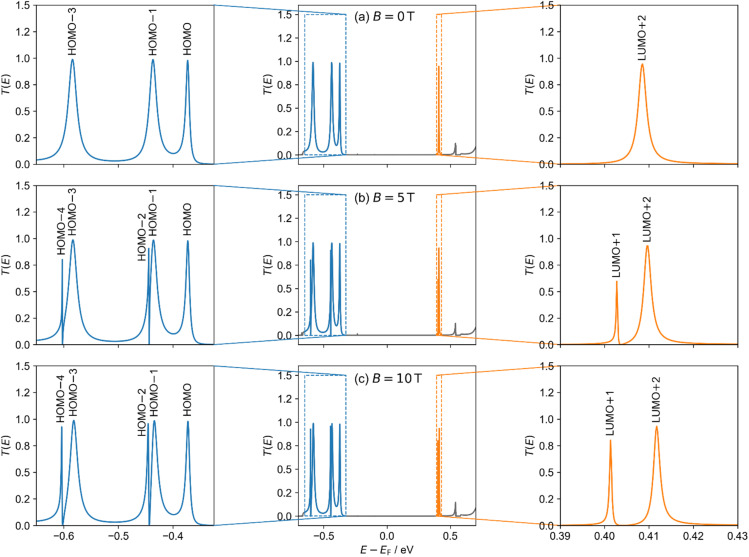
Transmittance probability *T*(*E*) (middle column) of the α-spin channel of the *c*-P10 molecular AB interferometer (Fig. 3c) under perpendicular magnetic fields of (a) 0, (b) 5, and (c) 10 T. Energies are shown relative to the HOMO–LUMO gap center of the isolated *c*-P10 molecular ring at 0 T. Left and right columns show a zoom-in on the occupied (blue) and virtual (orange) transmittance peaks, respectively. Transmission probability peaks are labelled with the corresponding isolated *c*-P10 ring orbital (see ESI Section S.8†). The β-spin channel shows only minor differences due to the spin-Zeeman interaction (see ESI Section S.7†).

We performed transport calculations using our NEGF+GFN1-xTB-M1 approach for *c-*P10 coupled to two polyacene leads *via meso*-acetylene-linkers positioned in a “*para*-type” configuration (see Fig. 3c) under finite magnetic fields. The α-spin channel electronic transmittance probability through the *c-*P10 ring junction as a function of the energy (measured with respect to the center of the HOMO–LUMO gap of the isolated ring at *B* = 0) is shown in Fig. 5 for three perpendicular magnetic field intensities: *B* = 0 T (Fig. 5a), *B* = 5 T (Fig. 5b), and *B* = 10 T (Fig. 5c).

In the absence of a magnetic field (Fig. 5a), the doubly degenerate LUMO+1 and LUMO+2 are split by 5 meV due to the lead coupling (see ESI Section S.8†) and only the LUMO+2 conducts current, as manifested by a single broadened Lorentzian-shaped transmittance peak (labelled LUMO+2 in Fig. 5a). At 5 T, another conductance channel opens *via* the second level, and destructive interference between the neighboring peaks induces an asymmetric shape (peaks labelled LUMO+1 and LUMO+2 in Fig. 5b). Increasing the magnetic field strength to 10 T further increases the inter-peak splitting, resulting in reduced interference and separated, nearly symmetric, Lorentzian-shaped peaks (labelled LUMO+1 and LUMO+2 in Fig. 5c). Similar responses are also seen for the HOMO−1 and HOMO−2 and the HOMO−3 and HOMO−4 pairs as shown in the left-hand side of Fig. 5. Since the peak pairs below the HOMO are considerably broader than those above the LUMO (indicating stronger coupling to the leads), highly asymmetric peaks appear once the additional channels open under a finite magnetic field. We identify these transmittance features as Fano resonances resulting from interference between states localized on the ring and delocalized over the whole device.

In contrast to the degenerate orbital pairs, the LUMO does not conduct current under the considered magnetic field strengths and is not associated with any transmittance peak. The HOMO, on the other hand, conducts current under all magnetic field strengths considered and is associated with a transmittance peak that remains unaffected by the magnetic field, except for a slight energy shift.

**Fig. 6:**
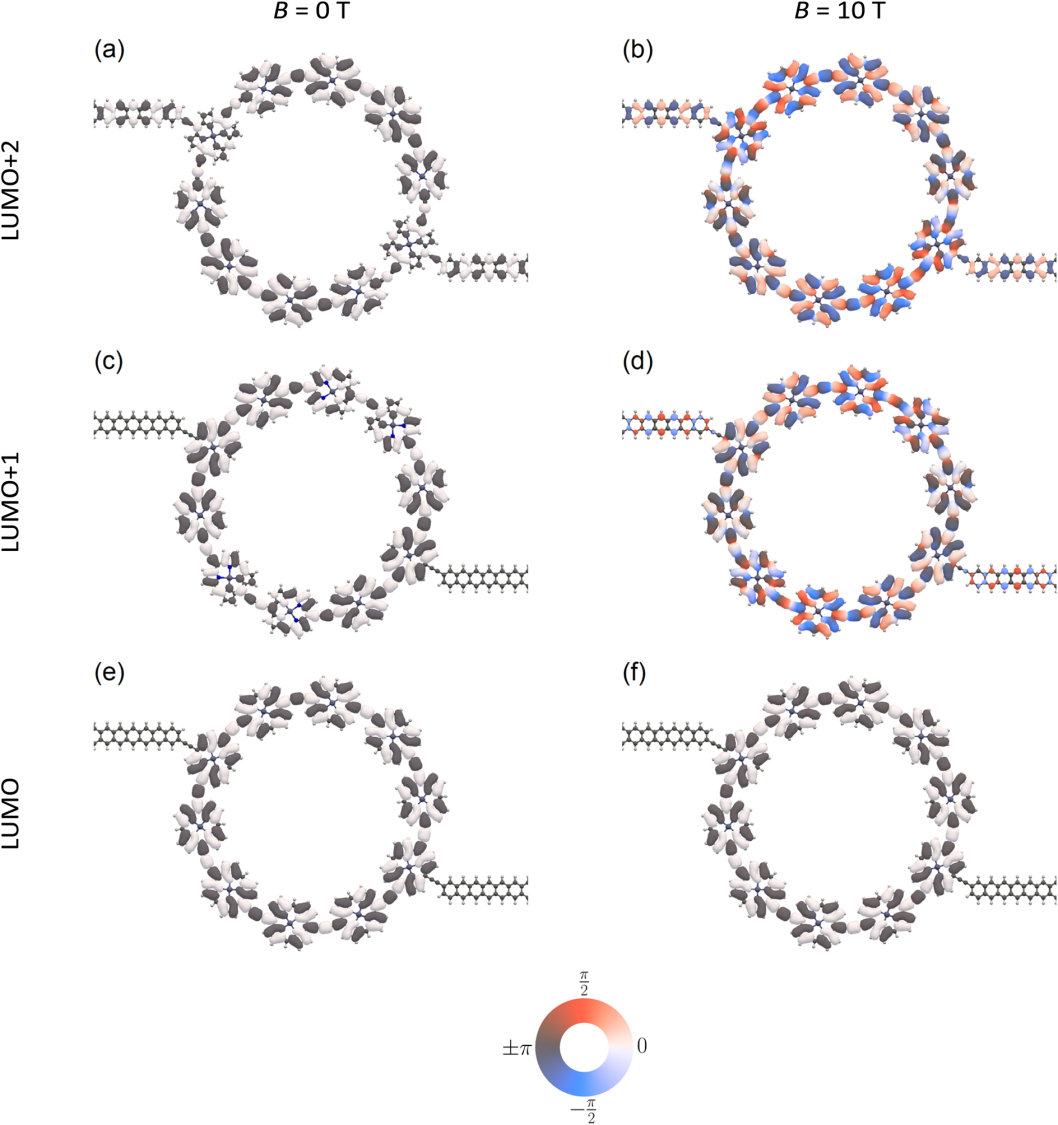
Representative molecular orbitals of a *c*-P10 junction in the absence (a, c and e) and the presence (b, d and f) of a 10 T magnetic field applied perpendicular to the plane of the ring. The junction orbitals (a and b), (c and d), and (e and f) correspond to the LUMO+2, LUMO+1 and LUMO of the isolated *c*-P10 molecular ring, respectively. The orbital plots are phase colored, so that purely dark gray and light gray (red and blue) colored orbitals correspond to purely real (imaginary) values, as indicated on the color wheel.

The results presented above can be rationalized by analyzing the relevant molecular orbitals in a finite junction model. For example, we can look at the junction orbitals corresponding to the LUMO+2, LUMO+1, and LUMO of the isolated ring. At *B* = 0, the LUMO+2 of the isolated ring delocalizes over the leads (Fig. 6a) yielding a continuous density across the junction that is manifested as an open channel in the transmittance probability curves. The LUMO+1 and LUMO are fully localized on the ring (Fig. 6c and e), exhibiting no probability density on the acetylene linkers and the adjacent polyacene lead sections, which explains why these orbitals do not carry current. When a 10 T field is applied, the LUMO+2 undergoes a phase shift, but the orbital remains delocalized over the junction (Fig. 6b) and therefore is still conductive. The previously ring-localized LUMO+1 now delocalizes onto the leads (Fig. 6d) with a similar phase shift, yielding a continuous density across the junction that is manifested as a new open channel in the transmittance probability. For the LUMO, no significant changes to the orbital are observed (Fig. 6f), and it remains non-conductive.

**Fig. 7:**
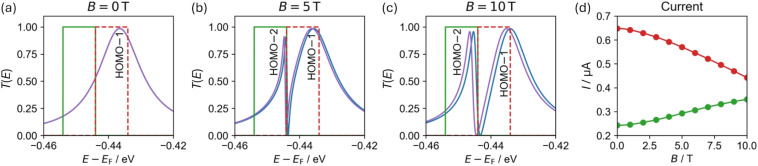
(a–c) Transmittance probability function for α (blue) and β (purple) spin channels in the vicinity of the HOMO−1 and HOMO−2 (see Fig. 5), calculated for a *c*-P10 junction under a perpendicular magnetic field of (a) 0, (b) 5, and (c) 10 T. (d) Magnetic field dependence of the current, calculated using eqn (6) with 10 meV wide Fermi windows placed around the HOMO−1 and HOMO−2 (red and green), respectively. Transmission probability peaks are labelled with the *c*-P10 ring orbital they correspond to (see ESI Section S.8†). Results with a 50 meV wide Fermi window show similar qualitative behavior (see ESI Section S.10†).

The sharp Fano resonances seen in Fig. 5 can be harnessed to modulate the current with accessible magnetic fields. To demonstrate this, we placed 10 meV wide Fermi transport windows around the transmittance peaks marked as HOMO−1 and HOMO−2 (gated to −449 and −439 meV, respectively, as marked by the colored green and red rectangles in Fig. 7a–c) and evaluated the current under various perpendicular magnetic fields using eqn (6). Fig. 7d shows the field dependence of the current, demonstrating that zero-temperature magnetoresistance can be achieved resulting in current variations of up to ∼32–45% at an experimentally accessible magnetic field of 10 T. Notably, by controlling the position of the Fermi transport window using a gate potential one can dictate whether the molecular interferometer will exhibit increased (green) or decreased (red) currents in response to the application of an external perpendicular magnetic field.

## 4 Conclusions

Molecular interferometry offers a unique route for controlling and manipulating the transport behavior and response properties of quantum electronic and spintronic nanodevices. Here, we have demonstrated that molecular AB interferometers based on synthetically available *c-*P10 porphyrin nanorings can be realized under realistic (∼10 T) magnetic fields. Using a newly developed semi-empirical computational approach for steady-state electronic transport calculations under finite magnetic fields, we have identified several important principles for designing a molecular AB interferometer: (i) the candidate molecular rings should be sufficiently large to allow for significant magnetic response at feasible field intensities, but not so large as to lose coherent transport; (ii) the chemical nature of the molecule should support a low effective mass of the conducting electrons to increase the state energy response towards the external magnetic field; and (iii) weak ring-lead couplings are required to allow for sharp transmittance features that increase the sensitivity of the current to the applied magnetic field. We calculated the effective mass, m*, of electrons in the molecular rings by fitting the variation in their orbital energies, as a function of magnetic field, to the PIR model. This analysis shows that butadiyne-linked nanorings, *c*-P*N*, have larger effective masses than edge-fused porphyrin nanobelts, *f*-P*N*. On the other hand, *c*-P*N*s have larger radii than *f*-P*N*s, for a given value of *N*, and *c*-P*N*s are more synthetically accessible.^59,60^ For the *c*-P10 molecular ring, we predict the emergence of Fano resonances at finite fields, resulting from field-induced channel openings. At low temperatures, these sharp transmittance features can be harnessed for sensitive interferometry and current switching. At higher electronic temperatures, the broadened Fermi transport window will result in reduced magneto-sensitivity (see ESI Section S.10†). In the model used here, the nanoring is weakly coupled to covalently attached polyacene leads, *via meso*-acetylene-linkers. An alternative design strategy for reducing the ring-lead couplings to achieve narrower transmittance peaks and higher magneto-sensitivity, would be to use weakly coupled non-covalent stacking interactions to graphene leads.^30^ In this study, we have focused on an AB interferometer derived from *c-*P10 because it is realistic to synthesize this nanoring with suitable substituents for attaching electrodes.^59,60^ On the other hand, the results in Table 2 imply that other molecular rings, such as *c-*P40, *f-*P30 and *f-*P40 would give even more sensitive interferometers.

Most previously reported magnetoresistance devices are based on paramagnetic systems, *i.e.* spin valves.^61–64^ The principles formulated here for designing diamagnetic molecular magnetoresistance devices are not limited to the *c-*P*N* family and are expected to be manifested in other molecular rings, provided they have large radii and charge carriers with small effective masses. For example, toroidal carbon nanotubes^55,56^ and charged polycyclic systems^65^ are promising candidates as AB interferometers. This work opens the door for the exploration of a variety of chemical and physical factors, such as the chemical nature of the molecular ring, the identity and position of the linkers, and the character of the metallic leads, which can be tuned to optimize the magnetic response of the system towards the experimental realization of molecular AB interferometry.

## Data availability

The data that support the findings of this study are available in the ESI† of this article.

## Author contributions

H. L. A. and O. H. initiated the project; C. Y. C. performed most of the computational work, supervised by A. M. W.-T.; geometries of porphyrin nanorings and nanobelts were calculated by I. R.; a draft manuscript was written by O. H., C. Y. C., and I. R.; G. H., O. H., and J. E. P. provided an initial NEGF code and participated in discussions regarding the code development and in the early stages of the calculations. All authors discussed the results and contributed towards refining the manuscript.

## Conflicts of interest

There are no conflicts to declare.

## Supplementary Material

SC-OLF-D4SC07992B-s001
