# Correction: Molecular Aharonov–Bohm-type interferometers based on porphyrin nanorings

**DOI:** 10.1039/d5sc90237a

**Published:** 2025-11-05

**Authors:** Chi Y. Cheng, Gil Harari, Igor Rončević, Juan E. Peralta, Harry L. Anderson, Andrew M. Wibowo-Teale, Oded Hod

**Affiliations:** a School of Chemistry, University of Nottingham, University Park Nottingham NG7 2RD UK; b School of Chemistry, The Sackler Centre for Computational Molecular and Materials Science, Tel Aviv University Tel Aviv 6997801 Israel; c Department of Chemistry, University of Oxford, Chemistry Research Laboratory Oxford OX1 3TA UK; d Department of Physics, Central Michigan University Mount Pleasant MI 48859 USA

## Abstract

Correction for ‘Molecular Aharonov–Bohm-type interferometers based on porphyrin nanorings’ by Chi Y. Cheng *et al.*, *Chem. Sci.*, 2025, **16**, 4392–4401, https://doi.org/10.1039/D4SC07992B.

The authors regret that the units of radius in column 2 of Tables 1 and S.2. are incorrect in their published article and SI.

The units of radius are hereby corrected from nm to Å and the corrected tables are provided below. The original SI document has been updated.

**Table 1** Calculated radii, effective masses m* and magnetic fields corresponding to one flux quantum *B*_0_ for selected nanoring orbitals

**Table:** 

Nanoring	*r*/Å	m*/*m*_e_ (HOMO)	m*/*m*_e_ (LUMO)	*B*_0_/*T*
***c-*P10**	21.1	−0.1081	0.1055	296
***c-*P20**	42.8	−0.1083	0.1057	72
***c-*P40**	86.0	−0.1084	0.1057	18
***f-*P10**	13.3	−0.0134	0.0129	744
***f-*P20**	26.7	−0.0073	0.0073	185
***f-*P40**	53.3	−0.0059	0.0058	46

**Table S.2.** Calculated effective masses m* and magnetic fields corresponding to one flux quantum *B*_0_ for selected nanoring orbitals of the ***c*-PN** nanorings in the non-planar (cylindrical) conformation

**Table:** 

Nanoring	*r*/Å	m*/*m*_e_ (HOMO)	m*/*m*_e_ (LUMO)	*B*_0_/*T*
***c*-P10**	21.4	−0.1054	0.1029	287
***c*-P20**	43.0	−0.1076	0.1050	71
***c*-P40**	86.0	−0.1082	0.1055	18

The Royal Society of Chemistry apologises for these errors and any consequent inconvenience to authors and readers.

