# Supplementary material for: Molecular Aharonov–Bohm-type interferometers based on porphyrin nanorings
Source: Chem Sci. 2025 Jan 29;16(10):4392–401. doi: 10.1039/d4sc07992b (PMC11795298; doi:10.1039/d4sc07992b)
Supplement: SC-016-D4SC07992B-s001 [file SC-016-D4SC07992B-s001.pdf]

Supplementary Information (SI) for Chemical Science.
This journal is © The Royal Society of Chemistry 2025

Supplementary Information

Molecular Aharonov-Bohm Interferometers Based on Porphyrin-Nanorings

Chi Y. Cheng,[a] Gil Harari,[b] Igor Rončević,[c] Juan E. Peralta,[d] Harry L. Anderson,[c]* Andrew M. Wibowo-Teale[a,]* and Oded Hod[b]*

[a] School of Chemistry, University of Nottingham, University Park, Nottingham, NG7 2RD, UK

[b] School of Chemistry and The Sackler Centre for Computational Molecular and Materials Science, Tel Aviv University, Tel Aviv 6997801, Israel

[c] Department of Chemistry, University of Oxford, Chemistry Research Laboratory, Oxford, OX1 3TA, UK

[d] Department of Physics, Central Michigan University, Mount Pleasant, MI 48859, USA

Table of Contents

S.1	Benzene MO energies	S2
S.2	GFN1-xTB-M1	S3
S.3	The non-self-consistent Green's function approach	S5
S.4	The iterative procedure	S6
S.5	The non-self-consistent Green's function approach with a biased electron density	S10
S.6	Non-planar ***c*-P*N*** porphyrin nanoring orbital energies	S15
S.7	β-Spin results	S17
S.8	Extended molecule density of states peak assignment	S19
S.9	The finite junction model	S21
S.10	Current switching at higher bias and electronic temperature	S27
S.11	Band structure calculations on infinite periodic linear ribbons	S29
S.12	List of data files deposited on Zenodo	S30
S.13	References	S31

S.1 Benzene MO energies

In Fig. 2 of the main article, we plotted the energy eigen-values of the particle-in-a-ring (PIR) model and compared them to the MO energies of the benzene molecule, in both cases, energies were plotted as a function of an external uniform magnetic field perpendicular to the ring systems. MO energies of the benzene molecule were calculated with the GFN1-xTB-M1 method (see Section S.2 and Refs. 1 to 3) without the contributions from the spin-Zeeman effect since this is not included in the PIR model. To reduce the complexity of the benzene MO energy plots, only MOs with contributions from the p_z atomic orbitals were shown. For completeness, we include in Fig S.1 the benzene α and β-spin MO energies with the spin-Zeeman interaction for the benzene MOs which included contributions from the p_z atomic orbitals.

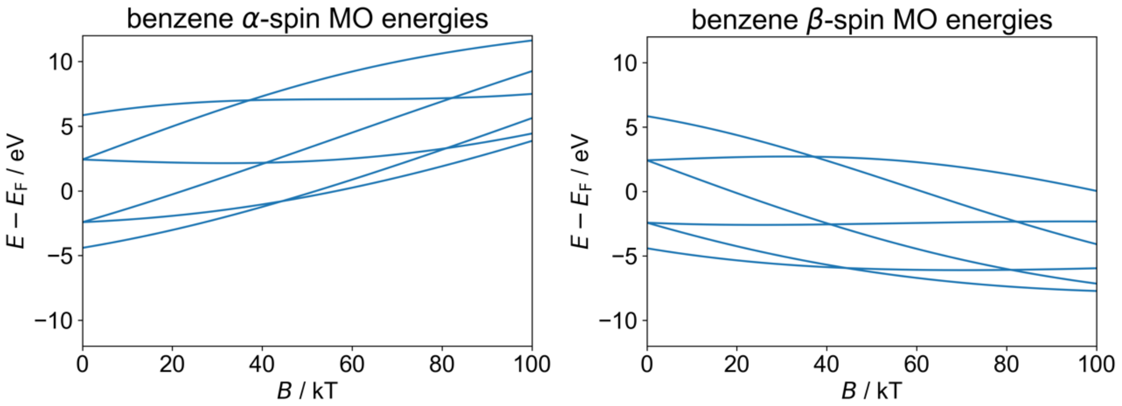

Fig. S.1. *Magnetic field dependence of the energy eigen-values of the benzene molecule (calculated using the GFN1-xTB-M1 method and plotted relative to the centre of the HOMO-LUMO gap in the absence of a magnetic field) only the benzene energy eigen-values of MOs with contributions from the* p_z *atomic orbitals are shown.*

S.2 GFN1-xTB-M1

All calculations presented in the main article were carried out using the GFN1-xTB-M1 variant of the GFN1-xTB-M extended tight-binding method,[1-3] which incorporates field-dependent kinetic and spin-Zeeman interaction energy terms in the Hamiltonian. To avoid gauge-origin dependencies of the calculations, the GFN1-xTB-M methods use London atomic orbitals (LAOs), which are obtained from standard atomic orbitals (AOs) through the multiplication of a field-dependent complex exponential:[4]

$$\omega_\nu(\mathbf{R}_\nu, \mathbf{r}) = \phi_\nu(\mathbf{R}_\nu, \mathbf{r})\exp\left(-\frac{ie}{\hbar}\mathbf{A}_\nu \cdot \mathbf{r}\right) \tag{1}$$

with

$$\mathbf{A}_\nu = \frac{1}{2}\mathbf{B} \times (\mathbf{R}_\nu - \mathbf{O}), \tag{2}$$

where $\omega_\nu(\mathbf{R}_\nu, \mathbf{r})$ are the LAOs, $\phi_\nu(\mathbf{R}_\nu, \mathbf{r})$ are AOs, $\mathbf{A}_\nu$ is the vector potential (defined in the Coulomb gauge) of a uniform magnetic field, $\mathbf{B}$, evaluated at the atomic orbital center $\mathbf{R}_\nu$ and $\mathbf{O}$ is the gauge-origin.

The use of LAOs leads to a reduction of the permutational symmetry of the two-electron integrals, which results in a greater computational burden that hampers the use of current density functional theory (CDFT) methods for large systems such as those considered herein. Notably, the GFN1-xTB-M1 method circumvents the need for explicit two-electron integral evaluations and considers only the valence electrons, resulting in a considerable reduction to the computational load. Further details of the GFN1-xTB-M1 method including comparisons against Hartree-Fock calculations of geometries, total energies, magnetic properties, and current densities under strong magnetic fields can be found in Ref. 3 below.

To validate the GFN1-xTB-M1 approach for the porphyrin nanorings considered in the main article, we compared the difference between the field-free and 5 T orbital energies of the non-planar ***c*-P4** and ***f*-P4** molecules calculated using GFN1-xTB-M1 and CDFT. For the CDFT calculations, we use the PBE0 exchange-correlation density functional with the 3-21G basis set and the resolution-of-the-identity approximation for the two-electron integral evaluations[5] with an auxiliary basis set generated using the AutoAux algorithm.[6] To that end, we used the optimized GFN1-xTB-M1 molecular geometries in the absence of a magnetic field.

First, we note that both GFN1-xTB-M1 and PBE0 provide a qualitatively similar low-energy spectrum, where the former yields smaller bandgaps and overall lower orbital energy spacings, as expected.[1] Table S.1 presents the change in the GFN1-xTB-M1 and PBE0 orbital energies upon the application of a perpendicular 5 T magnetic field. Here, as well, a qualitative agreement between the two methods is observed with some quantitative differences, where the GFN1-xTB-M1 generally underestimates the absolute value of the energy change occurring for the doubly degenerate orbitals for both the ***c*-P4** and ***f*-P4** nanorings. In the transmittance probability curves, a similar qualitative agreement is expected to occur with some quantitative differences in the peak positions. Given the good qualitative agreement obtained between both methods and the considerable computational burden associated with CDFT calculations, we have opted to use GFN1-xTB-M1 throughout the main article.

Spin	Orbital	ΔE / meV (GFN1-xTB-M1) *c*-**P4**	*f*-**P4**	ΔE / meV (PBE0/3-21G) *c*-**P4**	*f*-**P4**
α-spin	LUMO+3	0.29	0.29	0.29	0.29
	LUMO+2	1.67	1.06	2.41	2.20
	LUMO+1	−1.10	−0.48	−1.83	−1.63
	LUMO	0.29	0.29	0.29	0.30
	HOMO	0.29	0.29	0.28	0.28
	HOMO−1	1.62	0.29	2.50	0.29
	HOMO−2	−1.04	0.93	−1.92	1.35
	HOMO−3	0.29	−0.35	0.29	−0.77
β-spin	LUMO+3	−0.29	−0.29	−0.29	−0.29
	LUMO+2	1.10	0.48	1.83	1.63
	LUMO+1	−1.67	−1.06	−2.41	−2.21
	LUMO	−0.29	−0.28	−0.28	−0.28
	HOMO	−0.29	−0.29	−0.30	−0.29
	HOMO−1	1.04	−0.29	1.92	−0.29
	HOMO−2	−1.62	0.35	−2.50	0.77
	HOMO−3	−0.29	−0.93	−0.29	−1.35

Table S.1. *The change (with respect to the field-free case) in the α and β-spin orbital energies of the planar* ***c-P4*** *and the* ***f-P4*** *nanorings after a perpendicular magnetic field of 5 T is applied, calculated using GFN1-xTB-M1 and CDFT at the PBE0/3-21G level of theory.*

S.3 The non-self-consistent Green's function approach

To evaluate the current-voltage-magnetic-field characteristics of molecular interferometers, we implemented and applied a non-self-consistent non-equilibrium Green's function approach within the QUEST code. Given the overlap and effective Kohn-Sham matrix blocks of the extended molecule, the left and right leads, and their corresponding coupling matrices (evaluated in the absence of a bias voltage for the entire finite model system) we can obtain the necessary matrices required for the calculation of the transmission probability function via eq (8) in the main article. The dressed extended-molecule retarded Green's function is obtained from,

$$\mathbf{G}_{\mathrm{M}}^{\mathrm{r},\sigma}(E) = [(E + i\eta)\mathbf{S}_{\mathrm{M}} - \mathbf{F}_{\mathrm{M}}^{\sigma} - \mathbf{\Sigma}_{\mathrm{L}}^{\mathrm{r},\sigma}(E) - \mathbf{\Sigma}_{\mathrm{R}}^{\mathrm{r},\sigma}(E)]^{-1}, \tag{3}$$

where $\mathbf{F}_{\mathrm{M}}^{\sigma}$ and $\mathbf{S}_{\mathrm{M}}$ are the Kohn-Sham and overlap matrix blocks of the extended molecule section (including the molecular ring and its adjacent lead sections, chosen to be sufficiently long to avoid direct coupling between the ring and the leads), $\sigma = \alpha, \beta$ represents the spin state, and η is a small real number introduced to shift the poles of the Green's function from the real axis. The retarded lead self-energies are given by,

$$\mathbf{\Sigma}_{\mathrm{J=L/R}}^{\mathrm{r},\sigma}(E) = \left[(E + i\eta)\mathbf{S}_{\mathrm{MJ}} - \mathbf{F}_{\mathrm{MJ}}^{\sigma}\right]\mathbf{G}_{\mathrm{J}}^{\mathrm{r},\sigma}(E)\left[(E + i\eta)\mathbf{S}_{\mathrm{JM}} - \mathbf{F}_{\mathrm{JM}}^{\sigma}\right], \tag{4}$$

where $\mathbf{F}_{\mathrm{MJ}}^{\sigma}$ and $\mathbf{F}_{\mathrm{JM}}^{\sigma}$ ($\mathbf{S}_{\mathrm{MJ}}$ and $\mathbf{S}_{\mathrm{JM}}$) are the Kohn-Sham (overlap) block matrices between the left or right leads and the extended molecule, respectively. The retarded surface Green's functions of the bare left or right leads, $\mathbf{G}_{\mathrm{L/R}}^{\mathrm{r},\sigma}(E)$, are evaluated via an iterative procedure outlined in Refs. 7 and 8. Due to the presence of a finite magnetic field, this procedure must be modified to account for the fact that the Kohn-Sham and overlap matrix elements in the LAO basis are not necessarily translationally invariant (see Section S.4). The advanced lead self-energy is given by $\mathbf{\Sigma}_{\mathrm{L/R}}^{\mathrm{a},\sigma}(E) = \left[\mathbf{\Sigma}_{\mathrm{L/R}}^{\mathrm{r},\sigma}(E)\right]^{\dagger}$ and the broadening matrices appearing in eq (8) in the main article are obtained via,

$$\mathbf{\Gamma}_{\mathrm{L/R}}^{\sigma}(E) = i\left[\mathbf{\Sigma}_{\mathrm{L/R}}^{\mathrm{r},\sigma}(E) - \mathbf{\Sigma}_{\mathrm{L/R}}^{\mathrm{a},\sigma}(E)\right] \tag{5}$$

S.4 The iterative procedure

Due to the presence of a finite magnetic field, we outline some modifications that we made to the iterative procedure used to calculate the surface and bulk Green's functions (GF) of the semi-infinite leads. The original scheme, found in Refs. 7 and 8, divides a (semi-)infinite system into a set of principal layers, such that the inter-layer coupling is negligible beyond nearest neighbors. The approach progressively adds to the Green's function of the surface principal layer the contribution of increasingly remote bulk layers up to convergence to a given accuracy threshold.

S.4.1 The surface Green's function

In the absence of a magnetic field, the corresponding procedure to obtain the energy dependent surface Green's function $\mathbf{G}_{00}(E)$ follows the equation:

$$\begin{pmatrix} \varepsilon\mathbf{S}_{00}-\mathbf{H}_{00} & \varepsilon\mathbf{S}_{01}-\mathbf{V}_{01} & \mathbf{0} & \cdots \\ \varepsilon\mathbf{S}_{10}-\mathbf{V}_{10} & \varepsilon\mathbf{S}_{11}-\mathbf{H}_{11} & \varepsilon\mathbf{S}_{12}-\mathbf{V}_{12} & \cdots \\ \mathbf{0} & \varepsilon\mathbf{S}_{21}-\mathbf{V}_{21} & \varepsilon\mathbf{S}_{22}-\mathbf{H}_{22} & \cdots \\ \vdots & \vdots & \vdots & \ddots \end{pmatrix}\begin{pmatrix} \mathbf{G}_{00}(E) & \mathbf{G}_{01}(E) & \mathbf{G}_{02}(E) & \cdots \\ \mathbf{G}_{10}(E) & \mathbf{G}_{11}(E) & \mathbf{G}_{12}(E) & \cdots \\ \mathbf{G}_{20}(E) & \mathbf{G}_{21}(E) & \mathbf{G}_{22}(E) & \cdots \\ \vdots & \vdots & \vdots & \ddots \end{pmatrix} = \mathbb{I}. \quad (6)$$

If the overlap ($\mathbf{S}$), Hamiltonian ($\mathbf{H}$), and coupling ($\mathbf{V}$) matrix blocks for each principal layer and between each pair of adjacent layers beyond the surface layer are sufficiently similar, eq (6) can be approximated by,

$$\begin{pmatrix} \varepsilon\mathbf{S}_{00}-\mathbf{H}_{00} & \varepsilon\mathbf{S}_{01}-\mathbf{V}_{01} & \mathbf{0} & \cdots \\ \varepsilon\mathbf{S}_{10}-\mathbf{V}_{10} & \varepsilon\mathbf{S}_{11}-\mathbf{H}_{11} & \varepsilon\mathbf{S}_{01}-\mathbf{V}_{01} & \cdots \\ \mathbf{0} & \varepsilon\mathbf{S}_{10}-\mathbf{V}_{10} & \varepsilon\mathbf{S}_{11}-\mathbf{H}_{11} & \cdots \\ \vdots & \vdots & \vdots & \ddots \end{pmatrix}\begin{pmatrix} \mathbf{G}_{00}(E) & \mathbf{G}_{01}(E) & \mathbf{G}_{02}(E) & \cdots \\ \mathbf{G}_{10}(E) & \mathbf{G}_{11}(E) & \mathbf{G}_{12}(E) & \cdots \\ \mathbf{G}_{20}(E) & \mathbf{G}_{21}(E) & \mathbf{G}_{22}(E) & \cdots \\ \vdots & \vdots & \vdots & \ddots \end{pmatrix} = \mathbb{I}, \quad (7)$$

where $\mathbf{H}_{00}$ and $\mathbf{S}_{00}$ are the surface layer Hamiltonian and overlap block matrices, $\mathbf{H}_{11}$ and $\mathbf{S}_{11}$ are the bulk layer Hamiltonian and overlap block matrices, $\mathbf{S}_{10}/\mathbf{S}_{01}$ and $\mathbf{V}_{10}/\mathbf{V}_{01}$ are the overlap and coupling block matrices between adjacent layers respectively, and $\varepsilon = (E + i\eta)$. We deviate slightly from the original iterative scheme in Refs. 7 and 8 by allowing the surface layer to be different from the bulk layers. To obtain the electronic structure of the (semi-)infinite system using matrix blocks extracted from manageable finite system calculations via the following chain of equations:

$$\begin{aligned} (\varepsilon\mathbf{S}_{00}-\mathbf{H}_{00})\mathbf{G}_{00}(E) &= \mathbf{I} - (\varepsilon\mathbf{S}_{01}-\mathbf{V}_{01})\mathbf{G}_{10}(E) \\ (\varepsilon\mathbf{S}_{11}-\mathbf{H}_{11})\mathbf{G}_{10}(E) &= (\mathbf{V}_{10}-\varepsilon\mathbf{S}_{10})\mathbf{G}_{00}(E) + (\mathbf{V}_{01}-\varepsilon\mathbf{S}_{01})\mathbf{G}_{20}(E) \\ (\varepsilon\mathbf{S}_{11}-\mathbf{H}_{11})\mathbf{G}_{20}(E) &= (\mathbf{V}_{10}-\varepsilon\mathbf{S}_{10})\mathbf{G}_{10}(E) + (\mathbf{V}_{01}-\varepsilon\mathbf{S}_{01})\mathbf{G}_{30}(E), \\ &\vdots \end{aligned} \quad (8)$$

so that

$$\mathbf{G}_{00}(E) = [(\varepsilon\mathbf{S}_{00}-\mathbf{H}_{00}) + (\varepsilon\mathbf{S}_{01}-\mathbf{V}_{01})(\mathbf{t}_0 + \tilde{\mathbf{t}}_0\mathbf{t}_1 + \tilde{\mathbf{t}}_0\tilde{\mathbf{t}}_1\mathbf{t}_2 + \cdots)]^{-1}, \quad (9)$$

where

$$\begin{aligned} \mathbf{t}_0 &= (\varepsilon\mathbf{S}_{11}-\mathbf{H}_{11})^{-1}(\mathbf{V}_{10}-\varepsilon\mathbf{S}_{10}) \\ \tilde{\mathbf{t}}_0 &= (\varepsilon\mathbf{S}_{11}-\mathbf{H}_{11})^{-1}(\mathbf{V}_{01}-\varepsilon\mathbf{S}_{01}) \end{aligned} \quad (10)$$

and

$$
\begin{aligned}
\mathbf{t}_i &= (\mathbf{I} - \mathbf{t}_{i-1}\tilde{\mathbf{t}}_{i-1} - \tilde{\mathbf{t}}_{i-1}\mathbf{t}_{i-1})^{-1}\mathbf{t}_{i-1}^2 \\
\tilde{\mathbf{t}}_i &= (\mathbf{I} - \mathbf{t}_{i-1}\tilde{\mathbf{t}}_{i-1} - \tilde{\mathbf{t}}_{i-1}\mathbf{t}_{i-1})^{-1}\tilde{\mathbf{t}}_{i-1}^2.
\end{aligned} \tag{11}
$$

S.4.2 An iterative procedure for LAOs

When considering finite magnetic fields, the matrix blocks need to be written in the basis of LAOs rather than standard (e.g. Gaussian or Slater) atomic orbitals, to produce gauge-origin independent results.[4] The corresponding overlap matrix elements are then given by,

$$
S_{11\mu\nu} = \int \mathrm{d}\mathbf{r}\ e^{i(\mathbf{A}_\mu - \mathbf{A}_\nu)\cdot\mathbf{r}} \phi_\mu^*(\mathbf{R}_\mu, \mathbf{r}) \phi_\nu(\mathbf{R}_\nu, \mathbf{r}). \tag{12}
$$

Since in each iteration step, we are interested in adding the contribution of progressively displaced principal layers, the elements of the overlap matrices of the next two layers will be,

$$
\begin{aligned}
S_{22\mu\nu} &= \int \mathrm{d}\mathbf{r}\ e^{i(\mathbf{A}'_\mu - \mathbf{A}'_\nu)\cdot\mathbf{r}} \phi_\mu^*(\mathbf{R}'_\mu, \mathbf{r}) \phi_\nu(\mathbf{R}'_\nu, \mathbf{r}) \\
S_{33\mu\nu} &= \int \mathrm{d}\mathbf{r}\ e^{i(\mathbf{A}''_\mu - \mathbf{A}''_\nu)\cdot\mathbf{r}} \phi_\mu^*(\mathbf{R}''_\mu, \mathbf{r}) \phi_\nu(\mathbf{R}''_\nu, \mathbf{r}),
\end{aligned} \tag{13}
$$

where $\mathbf{R}'_\nu = \mathbf{R}_\nu + \mathbf{R}_\mathrm{T}$ and $\mathbf{R}''_\nu = \mathbf{R}_\nu + 2\mathbf{R}_\mathrm{T}$ are the positions of orbital ν in layers 2 and 3, respectively, $\mathbf{R}_\mathrm{T}$ is the translation vector, which relates one layer to another, and

$$
\begin{aligned}
\mathbf{A}'_\nu &= \frac{1}{2}\mathbf{B} \times (\mathbf{R}'_\nu - \mathbf{O}) = \frac{1}{2}\mathbf{B} \times (\mathbf{R}_\nu + \mathbf{R}_\mathrm{T} - \mathbf{O}) \\
\mathbf{A}''_\nu &= \frac{1}{2}\mathbf{B} \times (\mathbf{R}''_\nu - \mathbf{O}) = \frac{1}{2}\mathbf{B} \times (\mathbf{R}_\nu + 2\mathbf{R}_\mathrm{T} - \mathbf{O}).
\end{aligned} \tag{14}
$$

Since $\phi(\mathbf{R}, \mathbf{r})$ depends only on $(\mathbf{r} - \mathbf{R})$ we may write:

$$
\begin{aligned}
\phi_\nu(\mathbf{R}_\nu + \mathbf{R}_\mathrm{T}, \mathbf{r}) &= \phi_\nu(\mathbf{R}_\nu, \mathbf{r} - \mathbf{R}_\mathrm{T}) \\
\phi_\nu(\mathbf{R}_\nu + 2\mathbf{R}_\mathrm{T}, \mathbf{r}) &= \phi_\nu(\mathbf{R}_\nu, \mathbf{r} - 2\mathbf{R}_\mathrm{T}).
\end{aligned} \tag{15}
$$

Setting $\mathbf{r}' = \mathbf{r} - \mathbf{R}_\mathrm{T}$ and $\mathbf{r}'' = \mathbf{r} - \mathbf{2R}_\mathrm{T}$, we obtain the equations:

$$
\begin{aligned}
S_{22\mu\nu} &= e^{i(\mathbf{A}_\mu - \mathbf{A}_\nu)\cdot\mathbf{R}_\mathrm{T}} \int \mathrm{d}\mathbf{r}'\ e^{i(\mathbf{A}_\mu - \mathbf{A}_\nu)\cdot\mathbf{r}'} \phi_\mu^*(\mathbf{R}_\mu, \mathbf{r}') \phi_\nu(\mathbf{R}_\nu, \mathbf{r}') \\
S_{33\mu\nu} &= e^{2i(\mathbf{A}_\mu - \mathbf{A}_\nu)\cdot\mathbf{R}_\mathrm{T}} \int \mathrm{d}\mathbf{r}''\ e^{i(\mathbf{A}_\mu - \mathbf{A}_\nu)\cdot\mathbf{r}''} \phi_\mu^*(\mathbf{R}_\mu, \mathbf{r}'') \phi_\nu(\mathbf{R}_\nu, \mathbf{r}'').
\end{aligned} \tag{16}
$$

Since the integrations over $\mathbf{r}'$ and $\mathbf{r}''$ run over the same space as the integration over $\mathbf{r}$ in eq (12) the integrals are equivalent so that

$$
\begin{aligned}
S_{22\mu\nu} &= e^{i(\mathbf{A}_\mu - \mathbf{A}_\nu)\cdot\mathbf{R}_\mathrm{T}} S_{11\mu\nu} \\
S_{33\mu\nu} &= e^{2i(\mathbf{A}_\mu - \mathbf{A}_\nu)\cdot\mathbf{R}_\mathrm{T}} S_{11\mu\nu}.
\end{aligned} \tag{17}
$$

From eqs (17) we can see that we can obtain the block matrices $\mathbf{S}_{22}, \mathbf{S}_{33}, \cdots$ from $\mathbf{S}_{11}$ using the unitary matrix:

$$
\mathbf{U} = \begin{pmatrix} e^{i\mathbf{A}_\mu \cdot \mathbf{R}_\mathrm{T}} & 0 & 0 & \cdots \\ 0 & e^{i\mathbf{A}_\nu \cdot \mathbf{R}_\mathrm{T}} & 0 & \cdots \\ 0 & 0 & e^{i\mathbf{A}_\xi \cdot \mathbf{R}_\mathrm{T}} & \cdots \\ \vdots & \vdots & \vdots & \ddots \end{pmatrix}, \tag{18}
$$

so that,

$$
\begin{aligned}
\mathbf{S}_{22} &= \mathbf{U}\mathbf{S}_{11}\mathbf{U}^{-1} \\
\mathbf{S}_{33} &= \mathbf{U}\mathbf{S}_{22}\mathbf{U}^{-1} = \mathbf{U}^{2}\mathbf{S}_{11}\mathbf{U}^{-2}. \\
&\vdots
\end{aligned} \tag{19}
$$

The same transformation applies for the inter-layer overlap matrix blocks so, for example,

$$
\begin{aligned}
\mathbf{S}_{23} &= \mathbf{U}\mathbf{S}_{12}\mathbf{U}^{-1} \\
\mathbf{S}_{32} &= \mathbf{U}\mathbf{S}_{21}\mathbf{U}^{-1}. \\
&\vdots
\end{aligned} \tag{20}
$$

A similar derivation yields the same transformation for the layer Hamiltonian and inter-layer coupling matrix blocks.

The procedure adopted to construct the surface GF of the semi-infinite lead is based on an iterative construction of the GF generating equation eqs (8)–(11) generated from eq (7). In the presence of an external magnetic field, eq (7) should be modified according to eqs (19) and (20) such that,

$$
\begin{pmatrix} \varepsilon\mathbf{S}_{00} - \mathbf{H}_{00} & \varepsilon\mathbf{S}_{01} - \mathbf{V}_{01} & \mathbf{0} & \mathbf{0} & \cdots \\ \varepsilon\mathbf{S}_{10} - \mathbf{V}_{10} & \varepsilon\mathbf{S}_{11} - \mathbf{H}_{11} & \mathbf{U}(\varepsilon\mathbf{S}_{01} - \mathbf{V}_{01})\mathbf{U}^{-1} & \mathbf{0} & \cdots \\ \mathbf{0} & \mathbf{U}(\varepsilon\mathbf{S}_{10} - \mathbf{V}_{10})\mathbf{U}^{-1} & \mathbf{U}(\varepsilon\mathbf{S}_{11} - \mathbf{H}_{11})\mathbf{U}^{-1} & \mathbf{U}^{2}(\varepsilon\mathbf{S}_{01} - \mathbf{V}_{01})\mathbf{U}^{-2} & \cdots \\ \mathbf{0} & \mathbf{0} & \mathbf{U}^{2}(\varepsilon\mathbf{S}_{10} - \mathbf{V}_{10})\mathbf{U}^{-2} & \mathbf{U}^{2}(\varepsilon\mathbf{S}_{11} - \mathbf{H}_{11})\mathbf{U}^{-2} & \cdots \\ \vdots & \vdots & \vdots & \vdots & \ddots \end{pmatrix}
\times \begin{pmatrix} \mathbf{G}_{00}(E) & \mathbf{G}_{01}(E) & \mathbf{G}_{02}(E) & \mathbf{G}_{03}(E) & \cdots \\ \mathbf{G}_{10}(E) & \mathbf{G}_{11}(E) & \mathbf{G}_{12}(E) & \mathbf{G}_{13}(E) & \cdots \\ \mathbf{G}_{20}(E) & \mathbf{G}_{21}(E) & \mathbf{G}_{22}(E) & \mathbf{G}_{23}(E) & \cdots \\ \mathbf{G}_{30}(E) & \mathbf{G}_{31}(E) & \mathbf{G}_{32}(E) & \mathbf{G}_{33}(E) & \cdots \\ \vdots & \vdots & \vdots & \vdots & \ddots \end{pmatrix} = \mathbb{I}. \tag{21}
$$

While this equation could, in principle, be used to evaluate the surface GF, a more tractable version can be obtained by performing the following transformation,

$$
\mathbb{U}\begin{pmatrix} \varepsilon\mathbf{S}_{00} - \mathbf{H}_{00} & (\varepsilon\mathbf{S}_{01} - \mathbf{V}_{01})\mathbf{U} & \mathbf{0} & \mathbf{0} & \cdots \\ \mathbf{U}^{-1}(\varepsilon\mathbf{S}_{10} - \mathbf{V}_{10}) & \mathbf{U}^{-1}(\varepsilon\mathbf{S}_{11} - \mathbf{H}_{11})\mathbf{U} & (\varepsilon\mathbf{S}_{01} - \mathbf{V}_{01})\mathbf{U} & \mathbf{0} & \cdots \\ \mathbf{0} & \mathbf{U}^{-1}(\varepsilon\mathbf{S}_{10} - \mathbf{V}_{10}) & \mathbf{U}^{-1}(\varepsilon\mathbf{S}_{11} - \mathbf{H}_{11})\mathbf{U} & (\varepsilon\mathbf{S}_{01} - \mathbf{V}_{01})\mathbf{U} & \cdots \\ \mathbf{0} & \mathbf{0} & \mathbf{U}^{-1}(\varepsilon\mathbf{S}_{10} - \mathbf{V}_{10}) & \mathbf{U}^{-1}(\varepsilon\mathbf{S}_{11} - \mathbf{H}_{11})\mathbf{U} & \cdots \\ \vdots & \vdots & \vdots & \vdots & \ddots \end{pmatrix}\mathbb{U}^{-1}
\times \begin{pmatrix} \mathbf{G}_{00}(E) & \mathbf{G}_{01}(E) & \mathbf{G}_{02}(E) & \mathbf{G}_{03}(E) & \cdots \\ \mathbf{G}_{10}(E) & \mathbf{G}_{11}(E) & \mathbf{G}_{12}(E) & \mathbf{G}_{13}(E) & \cdots \\ \mathbf{G}_{20}(E) & \mathbf{G}_{21}(E) & \mathbf{G}_{22}(E) & \mathbf{G}_{23}(E) & \cdots \\ \mathbf{G}_{30}(E) & \mathbf{G}_{31}(E) & \mathbf{G}_{32}(E) & \mathbf{G}_{33}(E) & \cdots \\ \vdots & \vdots & \vdots & \vdots & \ddots \end{pmatrix} = \mathbb{I}, \tag{22}
$$

where the transformation matrix $\mathbb{U}$ reads as

$$\mathbb{U} = \begin{pmatrix} \mathbf{1} & \mathbf{0} & \mathbf{0} & \mathbf{0} & \cdots \\ \mathbf{0} & \mathbf{U} & \mathbf{0} & \mathbf{0} & \cdots \\ \mathbf{0} & \mathbf{0} & \mathbf{U}^2 & \mathbf{0} & \cdots \\ \mathbf{0} & \mathbf{0} & \mathbf{0} & \mathbf{U}^3 & \cdots \\ \vdots & \vdots & \vdots & \vdots & \ddots \end{pmatrix}. \tag{23}$$

Multiplying eq (22) from the left and right with $\mathbb{U}^{-1}$ and $\mathbb{U}$, respectively, we obtain

$$\begin{pmatrix} \varepsilon\mathbf{S}_{00} - \mathbf{H}_{00} & (\varepsilon\mathbf{S}_{01} - \mathbf{V}_{01})\mathbf{U} & \mathbf{0} & \mathbf{0} & \cdots \\ \mathbf{U}^{-1}(\varepsilon\mathbf{S}_{10} - \mathbf{V}_{10}) & \mathbf{U}^{-1}(\varepsilon\mathbf{S}_{11} - \mathbf{H}_{11})\mathbf{U} & (\varepsilon\mathbf{S}_{01} - \mathbf{V}_{01})\mathbf{U} & \mathbf{0} & \cdots \\ \mathbf{0} & \mathbf{U}^{-1}(\varepsilon\mathbf{S}_{10} - \mathbf{V}_{10}) & \mathbf{U}^{-1}(\varepsilon\mathbf{S}_{11} - \mathbf{H}_{11})\mathbf{U} & (\varepsilon\mathbf{S}_{01} - \mathbf{V}_{01})\mathbf{U} & \cdots \\ \mathbf{0} & \mathbf{0} & \mathbf{U}^{-1}(\varepsilon\mathbf{S}_{10} - \mathbf{V}_{10}) & \mathbf{U}^{-1}(\varepsilon\mathbf{S}_{11} - \mathbf{H}_{11})\mathbf{U} & \cdots \\ \vdots & \vdots & \vdots & \vdots & \ddots \end{pmatrix}$$
$$\times \mathbb{U}^{-1} \begin{pmatrix} \mathbf{G}_{00}(E) & \mathbf{G}_{01}(E) & \mathbf{G}_{02}(E) & \mathbf{G}_{03}(E) & \cdots \\ \mathbf{G}_{10}(E) & \mathbf{G}_{11}(E) & \mathbf{G}_{12}(E) & \mathbf{G}_{13}(E) & \cdots \\ \mathbf{G}_{20}(E) & \mathbf{G}_{21}(E) & \mathbf{G}_{22}(E) & \mathbf{G}_{23}(E) & \cdots \\ \mathbf{G}_{30}(E) & \mathbf{G}_{31}(E) & \mathbf{G}_{32}(E) & \mathbf{G}_{33}(E) & \cdots \\ \vdots & \vdots & \vdots & \vdots & \ddots \end{pmatrix} \mathbb{U} = \mathbb{I}. \tag{24}$$

We may now define the $\mathbb{U}$ transformed GF as follows

$$\mathbb{U}^{-1} \begin{pmatrix} \mathbf{G}_{00}(E) & \mathbf{G}_{01}(E) & \mathbf{G}_{02}(E) & \mathbf{G}_{03}(E) & \cdots \\ \mathbf{G}_{10}(E) & \mathbf{G}_{11}(E) & \mathbf{G}_{12}(E) & \mathbf{G}_{13}(E) & \cdots \\ \mathbf{G}_{20}(E) & \mathbf{G}_{21}(E) & \mathbf{G}_{22}(E) & \mathbf{G}_{23}(E) & \cdots \\ \mathbf{G}_{30}(E) & \mathbf{G}_{31}(E) & \mathbf{G}_{32}(E) & \mathbf{G}_{33}(E) & \cdots \\ \vdots & \vdots & \vdots & \vdots & \ddots \end{pmatrix} \mathbb{U} = \begin{pmatrix} \boldsymbol{\mathcal{G}}_{00}(E) & \boldsymbol{\mathcal{G}}_{01}(E) & \boldsymbol{\mathcal{G}}_{02}(E) & \boldsymbol{\mathcal{G}}_{03}(E) & \cdots \\ \boldsymbol{\mathcal{G}}_{10}(E) & \boldsymbol{\mathcal{G}}_{11}(E) & \boldsymbol{\mathcal{G}}_{12}(E) & \boldsymbol{\mathcal{G}}_{13}(E) & \cdots \\ \boldsymbol{\mathcal{G}}_{20}(E) & \boldsymbol{\mathcal{G}}_{21}(E) & \boldsymbol{\mathcal{G}}_{22}(E) & \boldsymbol{\mathcal{G}}_{23}(E) & \cdots \\ \boldsymbol{\mathcal{G}}_{30}(E) & \boldsymbol{\mathcal{G}}_{31}(E) & \boldsymbol{\mathcal{G}}_{32}(E) & \boldsymbol{\mathcal{G}}_{33}(E) & \cdots \\ \vdots & \vdots & \vdots & \vdots & \ddots \end{pmatrix}, \tag{25}$$

so that eq (24) becomes

$$\begin{pmatrix} \varepsilon\mathbf{S}_{00} - \mathbf{H}_{00} & (\varepsilon\mathbf{S}_{01} - \mathbf{V}_{01})\mathbf{U} & \mathbf{0} & \mathbf{0} & \cdots \\ \mathbf{U}^{-1}(\varepsilon\mathbf{S}_{10} - \mathbf{V}_{10}) & \mathbf{U}^{-1}(\varepsilon\mathbf{S}_{11} - \mathbf{H}_{11})\mathbf{U} & (\varepsilon\mathbf{S}_{01} - \mathbf{V}_{01})\mathbf{U} & \mathbf{0} & \cdots \\ \mathbf{0} & \mathbf{U}^{-1}(\varepsilon\mathbf{S}_{10} - \mathbf{V}_{10}) & \mathbf{U}^{-1}(\varepsilon\mathbf{S}_{11} - \mathbf{H}_{11})\mathbf{U} & (\varepsilon\mathbf{S}_{01} - \mathbf{V}_{01})\mathbf{U} & \cdots \\ \mathbf{0} & \mathbf{0} & \mathbf{U}^{-1}(\varepsilon\mathbf{S}_{10} - \mathbf{V}_{10}) & \mathbf{U}^{-1}(\varepsilon\mathbf{S}_{11} - \mathbf{H}_{11})\mathbf{U} & \cdots \\ \vdots & \vdots & \vdots & \vdots & \ddots \end{pmatrix}$$
$$\times \begin{pmatrix} \boldsymbol{\mathcal{G}}_{00}(E) & \boldsymbol{\mathcal{G}}_{01}(E) & \boldsymbol{\mathcal{G}}_{02}(E) & \boldsymbol{\mathcal{G}}_{03}(E) & \cdots \\ \boldsymbol{\mathcal{G}}_{10}(E) & \boldsymbol{\mathcal{G}}_{11}(E) & \boldsymbol{\mathcal{G}}_{12}(E) & \boldsymbol{\mathcal{G}}_{13}(E) & \cdots \\ \boldsymbol{\mathcal{G}}_{20}(E) & \boldsymbol{\mathcal{G}}_{21}(E) & \boldsymbol{\mathcal{G}}_{22}(E) & \boldsymbol{\mathcal{G}}_{23}(E) & \cdots \\ \boldsymbol{\mathcal{G}}_{30}(E) & \boldsymbol{\mathcal{G}}_{31}(E) & \boldsymbol{\mathcal{G}}_{32}(E) & \boldsymbol{\mathcal{G}}_{33}(E) & \cdots \\ \vdots & \vdots & \vdots & \vdots & \ddots \end{pmatrix} = \mathbb{I}. \tag{26}$$

The original iterative procedure can then be used to solve for $\boldsymbol{\mathcal{G}}_{00}(E)$, which according to eqs (23) and (25) satisfies $\boldsymbol{\mathcal{G}}_{00}(E) = \mathbf{G}_{00}(E)$. From eq (26) it becomes evident that the only modification required with respect to the field-free case is to perform the following block transformations

$$\begin{aligned} \varepsilon\mathbf{S}_{01} - \mathbf{V}_{01} &\to (\varepsilon\mathbf{S}_{01} - \mathbf{V}_{01})\mathbf{U} \\ \varepsilon\mathbf{S}_{10} - \mathbf{V}_{10} &\to \mathbf{U}^{-1}(\varepsilon\mathbf{S}_{10} - \mathbf{V}_{10}) \\ \varepsilon\mathbf{S}_{11} - \mathbf{H}_{11} &\to \mathbf{U}^{-1}(\varepsilon\mathbf{S}_{11} - \mathbf{H}_{11})\mathbf{U}, \end{aligned} \tag{27}$$

and use them in the original iterative scheme.

S.5 The non-self-consistent Green's function approach with a biased electron density

In this section, we compare the transmission functions of the ***c*-P10** molecular AB interferometer, which uses an equilibrium electron density, against those that use a biased electron density to mimic the effect of the electric field falling across the junction due to biased leads. To obtain an effective Kohn-Sham matrix with a biased electron density we run the GFN1-xTB-M1 calculation of a finite junction model under an error function electric potential

$$V_{\mathrm{erf}}(x) = \frac{1}{2} V_{\mathrm{b}} \,\mathrm{erf}\big(\lambda(x - x_0)\big), \quad (28)$$

where V_{b} is the bias between the leads, λ is a parameter that controls the rate at which the potential goes from $-V_{\mathrm{b}}/2$ to $V_{\mathrm{b}}/2$ and x_0 is a parameter that is used to center the potential, an example plot of this potential is shown in Fig. S.2.

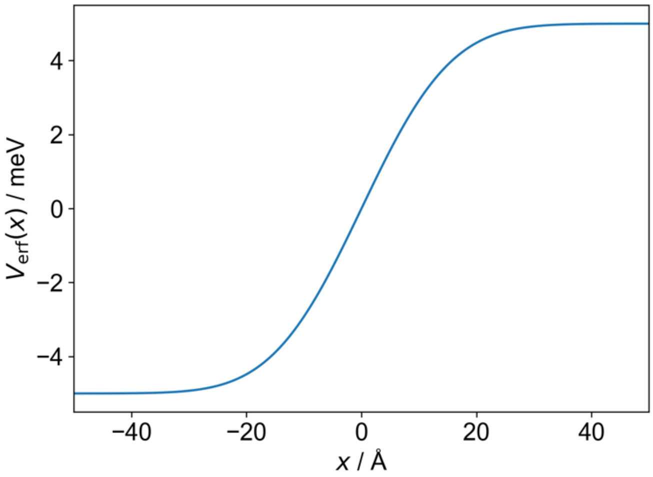

Fig. S.2. *An example error function potential with V_b = 10 meV, λ = 0.0575 Å^{-1} and x_0 = 0 Å, which is used to bias the electron density of a molecular AB interferometer.*

Before GFN1-xTB-M1 calculations are carried out, the reduced junction model must be rotated so that the acetylene-linkers lie along the x-axis and the error function potential is applied along the leads. With the error function potential, the self-consistent charge (SCC) iterations will result in charge polarization across the finite junction model. Once the SCC iterations converge, we can remove the error function potential from the resulting effective Kohn-Sham matrix to obtain the original Kohn-Sham matrix with a biased electron density, which will produce an effective bias potential across the extended molecule region of the junction. The Kohn-Sham matrix with the biased electron density is then used in the non-self-consistent NEGF approach to calculate the transmission function and compare against the results from the main article.

In Figs. S.3 to S.6, we show the transmission function with Kohn-Sham matrices obtained from the procedure described above using an error function potential with V_{b} = 10 and 100 meV, respectively, in both cases λ = 0.0575 Å^{-1} and x_0 was used to center the potential to the middle of the ***c*-P10** ring. With the smaller (10 meV) bias, the transmission function is essentially indistinguishable from the results presented in the main article.

For the larger (100 meV) bias, only small quantitative differences can be seen for the transmission function peaks that we are interested in, specifically the HOMO−4, HOMO−3, HOMO−2, HOMO−1 and the LUMO+1 and LUMO+2 peaks. This justifies the use of the non-self-consistent NEGF procedure in our calculations.

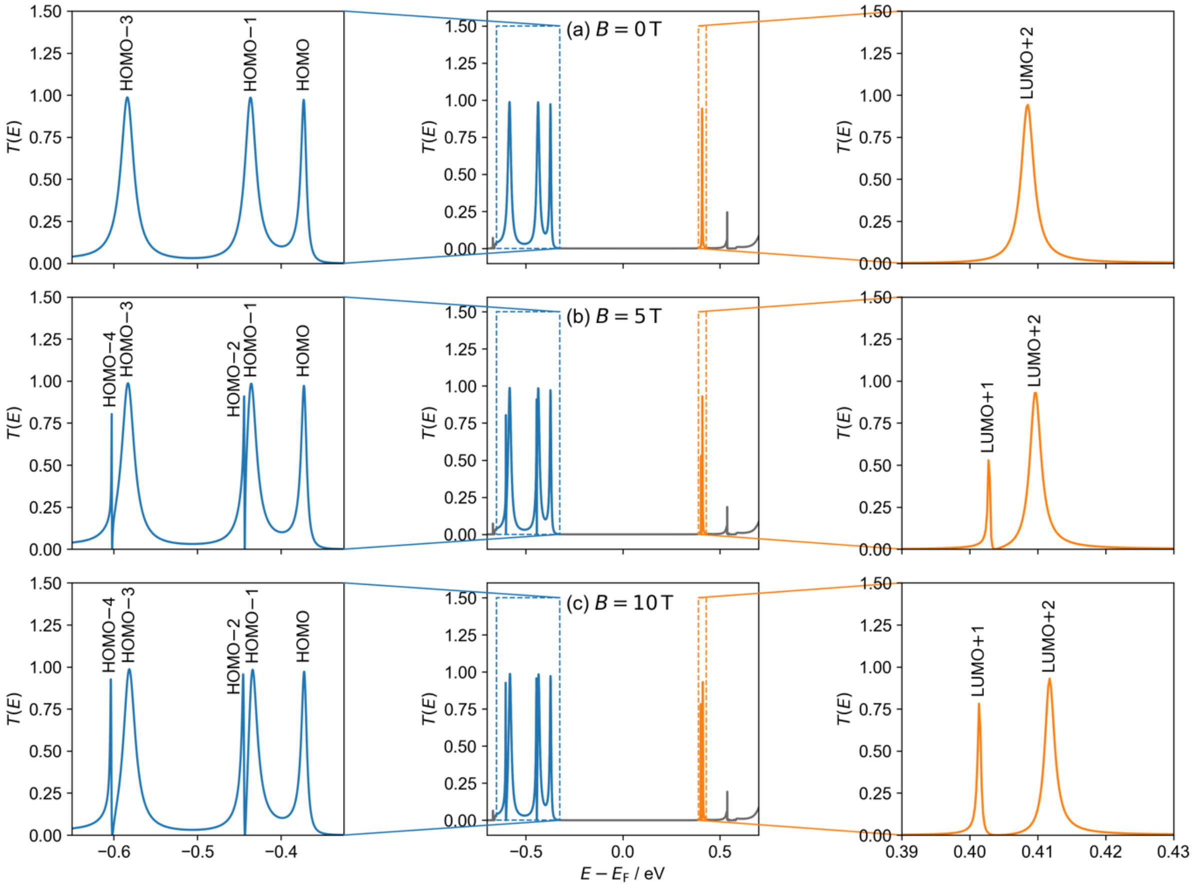

Fig. S.3. *Transmittance probability T(E) (middle column) of the α-spin channel of the* ***c-P10*** *molecular AB interferometer (Fig. 3c in the main article) with a biased electron density generated using the error function potential method described above with* $V_b = 10$ *meV,* $\lambda = 0.0575$ *Å*$^{-1}$*, and an* x_0 *value that centers the potential at the middle of the ring. Transmission probabilities were calculated under perpendicular magnetic fields of (a) 0, (b) 5, and (c) 10 T. Energies are shown relative to the HOMO-LUMO gap center of the isolated* ***c-P10*** *molecular ring at 0 T. Left and right columns show a zoom-in on the occupied (blue) and virtual (orange) transmittance peaks, respectively. Transmission probability peaks are labelled with the corresponding isolated* ***c-P10*** *ring orbital (see Section S.8).*

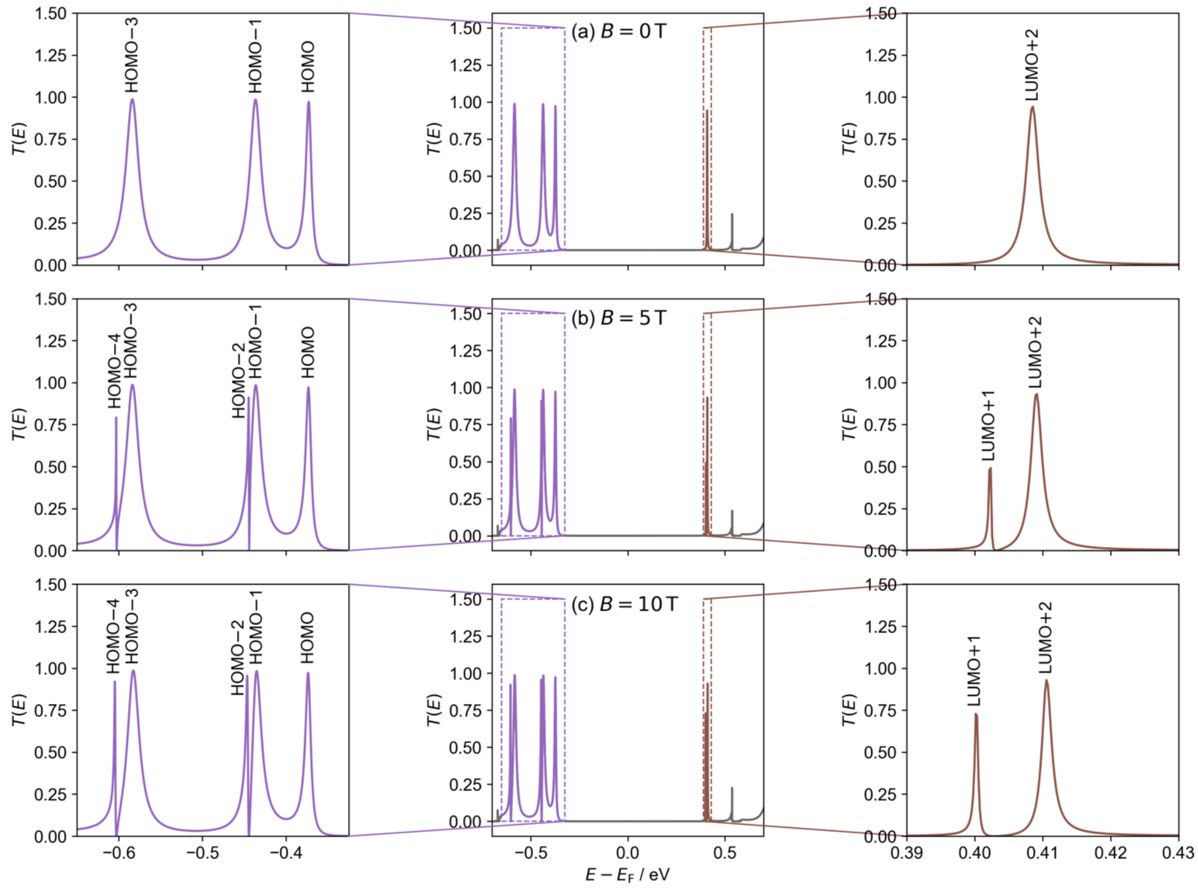

Fig. S.4. *Transmittance probability T(E) (middle column) of the β-spin channel of the* ***c-P10*** *molecular AB interferometer (Fig. 3c in the main article) with a biased electron density generated using the error function potential method described above with* V_b *= 10 meV, λ = 0.0575 Å*$^{-1}$*, and an* x_0 *value that centers the potential at the middle of the ring. Transmission probabilities were calculated under perpendicular magnetic fields of (a) 0, (b) 5, and (c) 10 T. Energies are shown relative to the HOMO-LUMO gap center of the isolated* ***c-P10*** *molecular ring at 0 T. Left and right columns show a zoom-in on the occupied (purple) and virtual (brown) transmittance peaks, respectively. Transmission probability peaks are labelled with the corresponding isolated* ***c-P10*** *ring orbital (see Section S.8).*

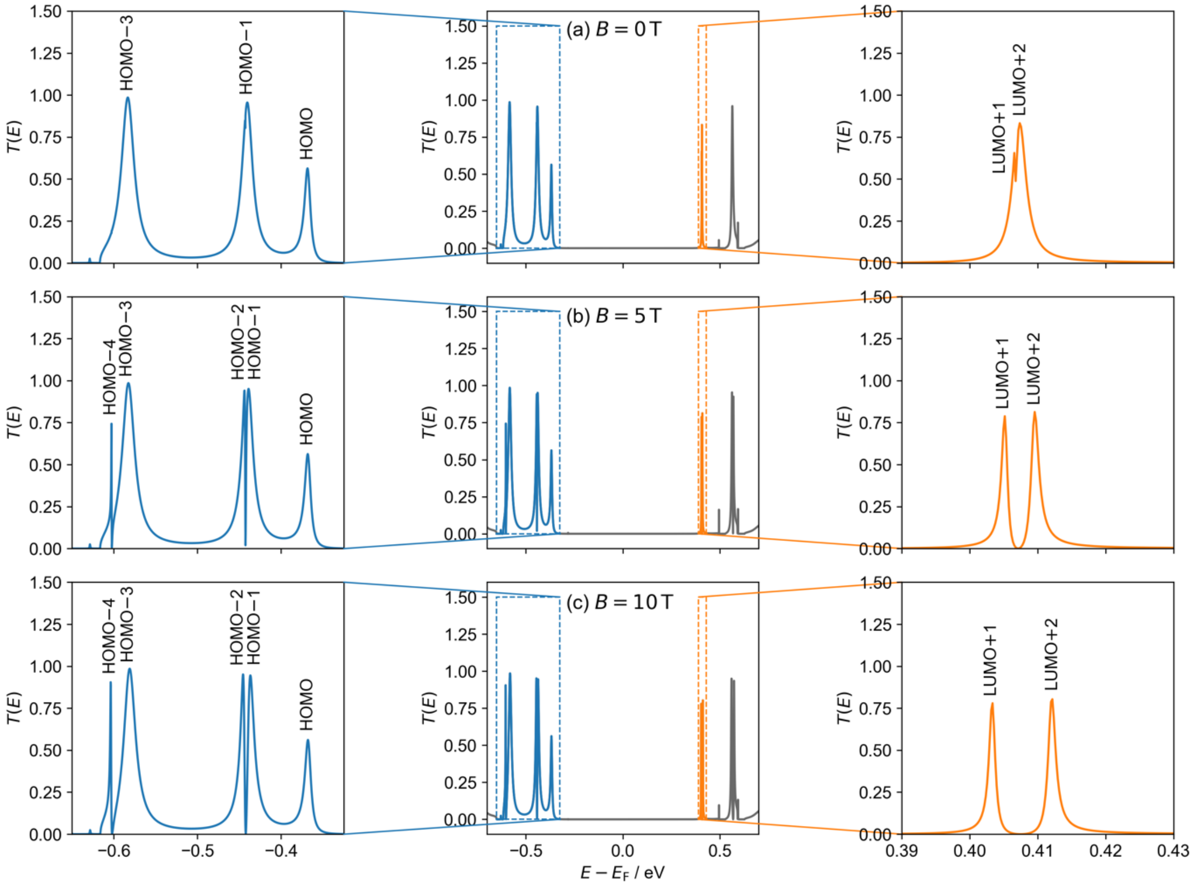

Fig. S.5. *Transmittance probability T(E) (middle column) of the α-spin channel of the* ***c-P10*** *molecular AB interferometer (Fig. 3c in the main article) with a biased electron density generated using the error function potential method described above with* $V_b = 100$ *meV,* $\lambda = 0.0575$ *Å*$^{-1}$*, and an* x_0 *value that centers the potential at the middle of the ring. Transmission probabilities were calculated under perpendicular magnetic fields of (a) 0, (b) 5, and (c) 10 T. Energies are shown relative to the HOMO-LUMO gap center of the isolated* ***c-P10*** *molecular ring at 0 T. Left and right columns show a zoom-in on the occupied (blue) and virtual (orange) transmittance peaks, respectively. Transmission probability peaks are labelled with the corresponding isolated* ***c-P10*** *ring orbital (see Section S.8).*

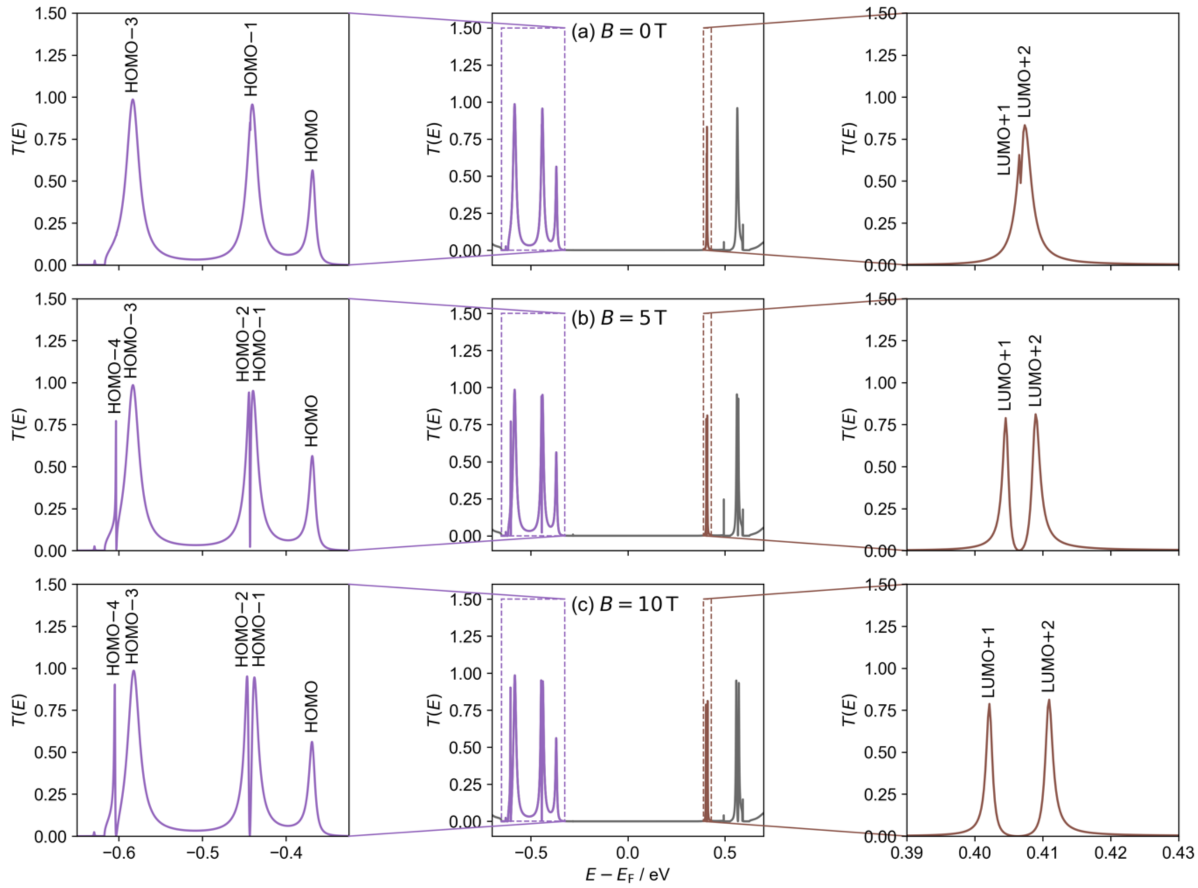

Fig. S.6. *Transmittance probability T(E) (middle column) of the β-spin channel of the* ***c-P10*** *molecular AB interferometer (Fig. 3c in the main article) with a biased electron density generated using the error function potential method described above with V_b = 100 meV, λ = 0.0575 Å^{-1}, and an x_0 value that centers the potential at the middle of the ring. Transmission probabilities were calculated under perpendicular magnetic fields of (a) 0, (b) 5, and (c) 10 T. Energies are shown relative to the HOMO-LUMO gap center of the isolated* ***c-P10*** *molecular ring at 0 T. Left and right columns show a zoom-in on the occupied (purple) and virtual (brown) transmittance peaks, respectively. Transmission probability peaks are labelled with the corresponding isolated* ***c-P10*** *ring orbital (see Section S.8).*

S.6 Non-planar ***c*-P*N*** porphyrin nanoring orbital energies

In the main text, we presented the orbital energies of a set of planar ***c*-P*N*** porphyrin nanorings. We focused on these conformers since they are expected to be more suitable for junction fabrication. For completeness, we report below the orbital energies for the corresponding lower energy non-planar ***c*-P*N*** molecular rings. Using GFN1-xTB-M1 we optimized non-planar ***c*-P*N*** porphyrin nanorings with 10, 20, 30, and 40 porphyrin monomer units in the absence of a magnetic field, yielding optimized ring radii of 21.4, 43.0, 64.5, and 86.0 Å, respectively (see Fig. S.7 for the optimized non-planar ***c*-P10** molecule). In Fig. S.8 we show the magnetic field dependence of α and β-spin molecular orbital (MO) energies and in Table S.2 the calculated effective masses for the HOMO and LUMO orbitals for non-planar ***c*-P*N***, demonstrating similarity to the planar counterparts (see Fig. 4 and Table 1 of the main text and Fig. S.9).

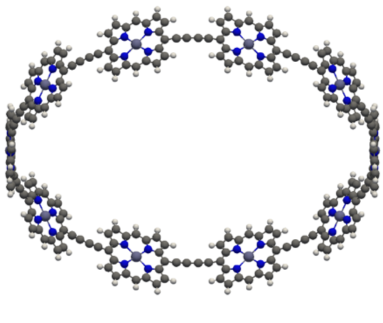

Fig. S.7. *GFN1-xTB optimized geometry of the non-planar **c-P10** porphyrin nanoring.*

Nanoring	r / nm	m^* / m_e (HOMO)	m^* / m_e (LUMO)	B_0 / T
***c*-P10**	21.4	−0.1054	0.1029	287
***c*-P20**	43.0	−0.1076	0.1050	71
***c*-P40**	86.0	−0.1082	0.1055	18

Table S.2. *Calculated effective masses m* and magnetic fields corresponding to one flux quantum B_0 for selected nanoring orbitals of the **c-PN** nanorings in the non-planar (cylindrical) conformation.*

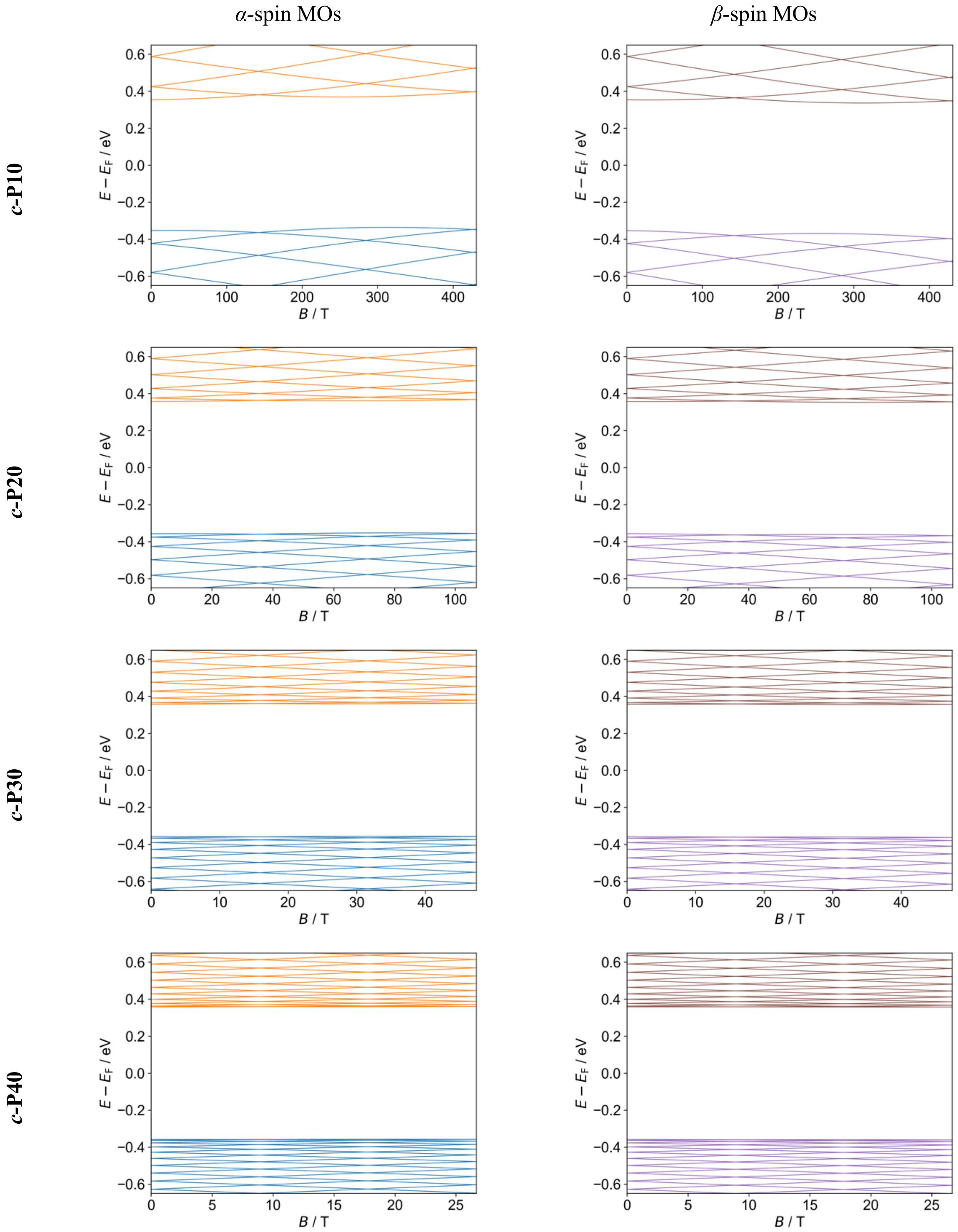

Fig. S.8. *GFN1-xTB-M1 non-planar* ***c-P10*** *(top row),* ***c-P20*** *(second row),* ***c-P30*** *(third row), and* ***c-P40*** *(bottom row) α (left) and β (right) spin orbital energies as a function of the perpendicular magnetic field intensity. Orbital energies are plotted relative to the corresponding HOMO-LUMO gap center in the absence of a magnetic field. Blue and orange colors are used for the α-spin occupied and unoccupied orbital energies, respectively, and purple and brown colors are used for the corresponding β-spin orbital energies.*

S.7 β-Spin results

In the main text, we presented α-spin results for the orbital energies of the planar ***c*-P*N*** and the ***f*-P*N*** nanoring sets, as well as the transmittance probability curves for the α-spin channel of the ***c*-P10** molecular AB interferometer. For completeness, we provide below the corresponding β-spin results. Overall, the results obtained for the β-spin are in qualitative agreement with those presented in the main text for the α-spin counterpart. Some minor quantitative differences arise at high magnetic field strengths, due to the spin-Zeeman interaction.

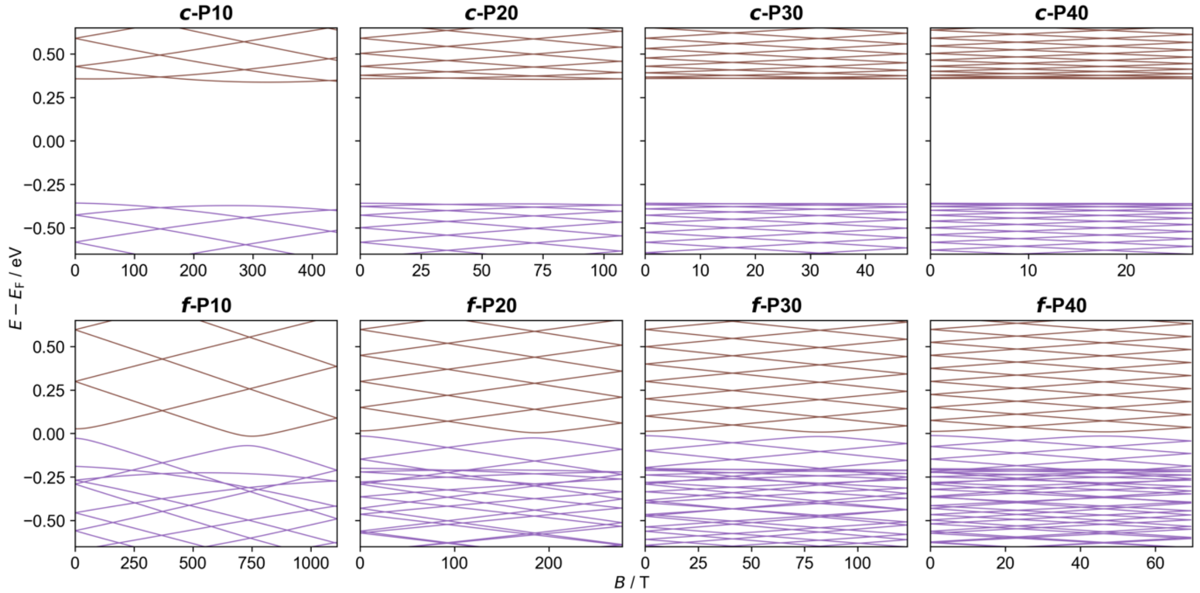

***Fig. S.9.** The β-spin orbital energies of planar **c-P10**, **c-P20**, **c-P30**, **c-P40** (upper panels) and **f-P10**, **f-P20**, **f-P30**, **f-P40** (lower panels) molecular rings as a function of the perpendicular magnetic field strength. Energies are plotted relative to the corresponding field-free HOMO-LUMO gap center. Occupied and unoccupied orbitals are shown in purple and brown, respectively.*

	ΔE / meV				ΔE / meV			
Orbital	***c*-P10**	***c*-P20**	***c*-P30**	***c*-P40**[a]	***f*-P10**	***f*-P20**	***f*-P30**	***f*-P40**
LUMO+4	5.9	8.7	10.1	11.1	3.4	7.5	11.6	15.6
LUMO+3	−7.0	−9.4	−9.9	−9.6	−4.6	−8.7	−12.7	−16.7
LUMO+2	4.0	4.9	5.6	6.3	3.5	7.5	11.4	15.4
LUMO+1	−5.1	−5.4	−5.1	−4.5	−4.6	−8.6	−12.5	−16.4
LUMO	−0.5	−0.2	0.3	1.0	−0.3	1.4	4.2	7.5
HOMO	−0.7	−1.0	−1.4	−2.1	−0.9	−2.6	−5.4	−8.6
HOMO−1	3.7	4.2	3.8	3.2	−0.6	7.2	11.2	15.1
HOMO−2	−5.0	−5.9	−6.6	−7.3	1.4	−8.4	−12.4	−16.3
HOMO−3	5.5	8.0	8.5	8.2	−2.6	−0.6	11.1	15.1
HOMO−4	−6.7	−9.6	−10.9	−11.9	3.2	1.6	−12.3[a]	−16.3

***Table S.3.** The change in the β-spin orbital energies ΔE (with respect to the field-free case) of the planar **c-P10**, **c-P20**, **c-P30**, **c-P40** and **f-P10**, **f-P20**, **f-P30**, **f-P40** nanorings due to a perpendicular magnetic field of 10 T. [a]For all **c-P40** orbitals and the **f-P30** HOMO−4, the 10 T magnetic field was sufficiently large to induce state crossing. In these cases, we calculate the energy changes by following the orbital through the crossing.*

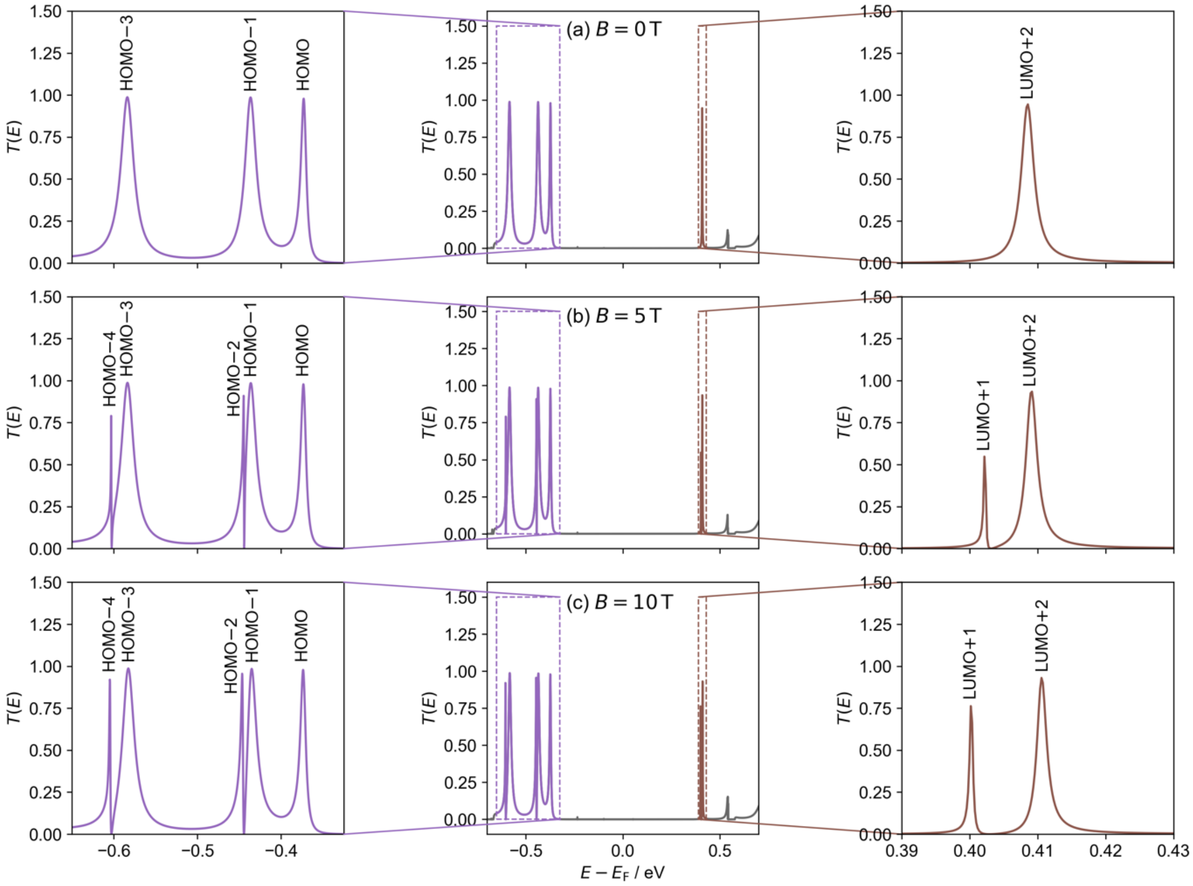

Fig. S.10. *Transmittance probability T(E) (middle column) of the β-spin channel of the **c-P10** molecular AB interferometer (Fig. 3c in the main article) under perpendicular magnetic fields of (a) 0, (b) 5, and (c) 10 T. Energies are shown relative to the HOMO-LUMO gap center of the isolated **c-P10** molecular ring at 0 T. Left and right columns show a zoom-in on the occupied (purple) and virtual (brown) transmittance peaks, respectively. Transmission probability peaks are labelled with the corresponding isolated **c-P10** ring orbital (see Section S.8).*

S.8 Extended molecule density of states peak assignment

In the main text, we considered both isolated and lead-coupled ***c*-P10** molecular rings. To compare the isolated system behavior to that of the coupled system, it is important to identify equivalent MOs. To that end, we plotted MOs of the two systems (an isolated ring and a finite junction model consisting of the ring, linkers, and hydrogen-passivated lead sections formed from 13 fused benzene rings) and matched specific isolated molecular ring orbitals with the corresponding MOs of the coupled system, based on spatial distribution (see Fig. S.15). The corresponding field-free orbital energies of the isolated ring and the finite junction model are presented in the left and center sub-tables of Table S.4, respectively. Next, we associate these eigen-states with peaks in the transmittance probability curve. To demonstrate how this is performed we plot in Figs. S.11 the density of states (DOS) of the infinite junction model, as evaluated from the extended molecule Green's function block in the presence of the semi-infinite leads surface Green's functions, which were obtained from the iterative scheme described in Section S.4. Focusing on, for example, the HOMO−8 and HOMO−7 of the finite junction model, their orbital energies match the peaks marked as 1 and 2 in the DOS diagram (compare the center and right sub-tables of Table S.4). In the corresponding transmittance probability curve at 0 T, a peak appears only at the energy of DOS peak 2, which is rationalized by the fact that the HOMO−8 is localized on the ring and does not present electron density along the linkers, whereas the HOMO−7 state delocalizes across the junction. The fact that at a finite magnetic field, a transmittance peak appears at the energy of DOS peak 1 and the HOMO−8 delocalizes across the junction, further supports the peak assignment.

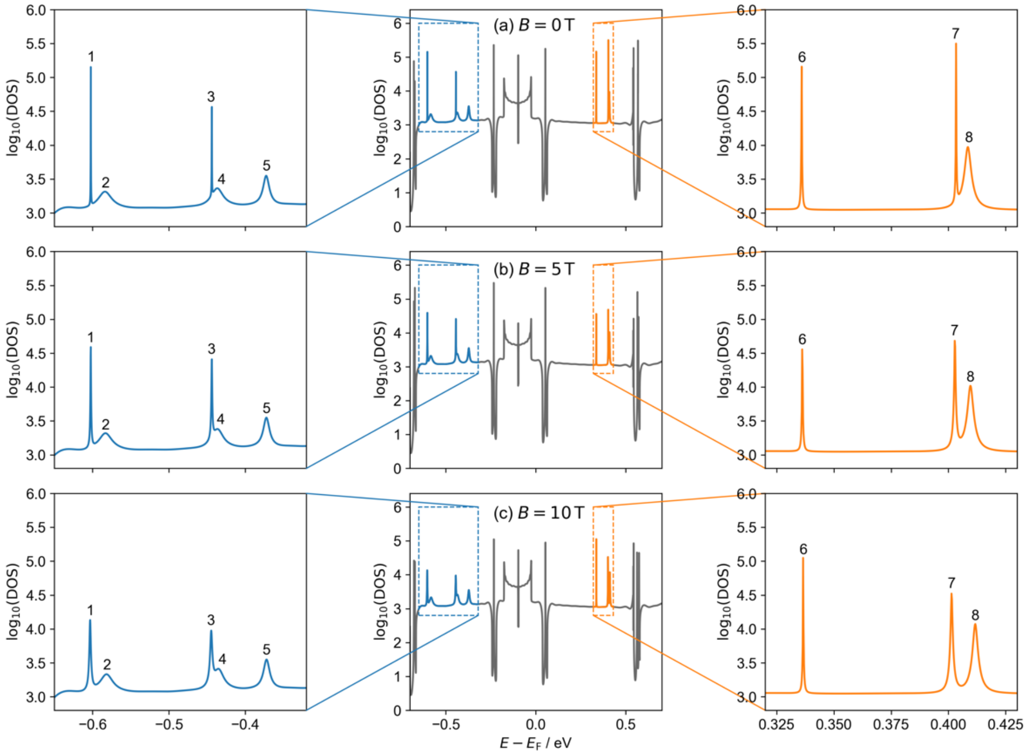

Fig. S.11. *DOS of the extended molecule section for the α-spin channel of the **c-P10** molecular AB interferometer (structure presented in Fig. 3c of the main text) under perpendicular magnetic fields of (a) 0, (b) 5, and (c) 10 T. Energies are presented relative to the HOMO-LUMO gap center of the isolated **c-P10** at 0 T. Left and right columns show sections of the occupied (blue) and virtual (orange) DOS, respectively.*

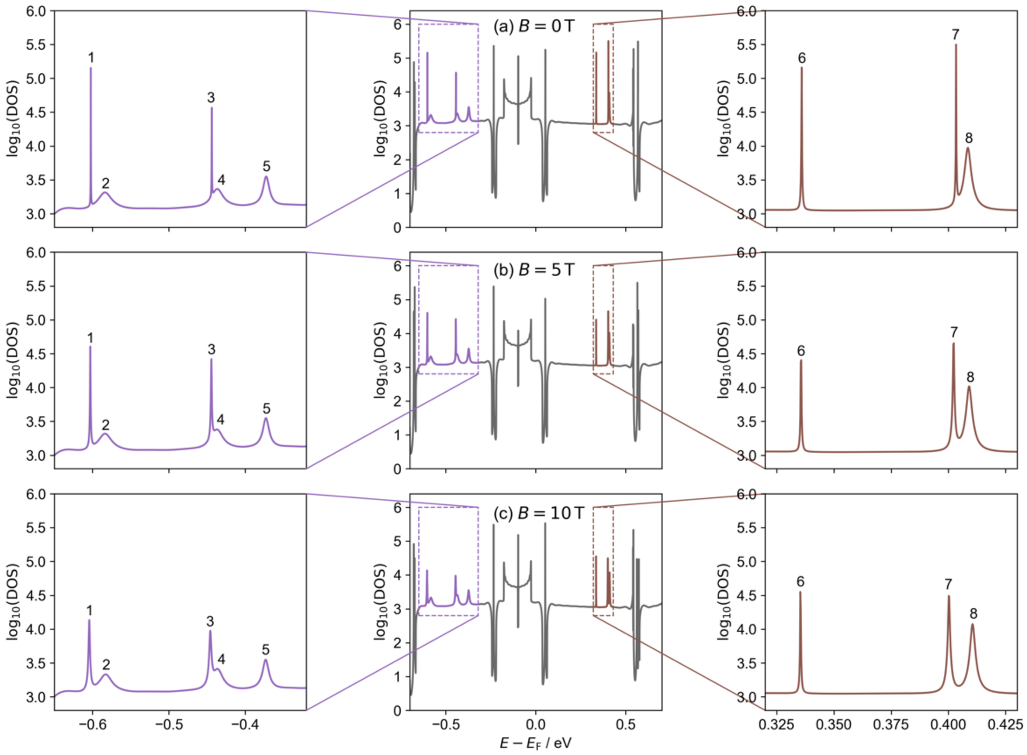

Fig. S.12. *DOS of the extended molecule section for the β-spin channel of the* ***c-P10*** *molecular AB interferometer (structure presented in Fig. 3c of the main text) under perpendicular magnetic fields of (a) 0, (b) 5, and (c) 10 T. Energies are presented relative to the HOMO-LUMO gap center of the isolated* ***c-P10*** *at 0 T. Left and right columns show sections of the occupied (purple) and virtual (brown) DOS, respectively.*

Isolated ***c*-P10** orbital energies		Finite junction orbital energies		DOS peak energies	
Orbital	$E - E_F$ / meV	Orbital	$E - E_F$ / meV	Peak	$E - E_F$ / meV
LUMO+2	428	LUMO+6	417	8	409
LUMO+1	428	LUMO+5	410	7	403
LUMO	357	LUMO+2	342	6	336
HOMO	−357	HOMO−2	−364	5	−373
HOMO−1	−426	HOMO−3	−416	4	−437
HOMO−2	−426	HOMO−4	−439	3	−444
HOMO−3	−582	HOMO−7	−588	2	−584
HOMO−4	−582	HOMO−8	−597	1	−602

Table S.4. *Tables of the isolated* ***c-P10*** *orbital energies (left), energies of the equivalent orbitals in the finite junction model (see Section S.9) consisting of the ring, linkers, and hydrogen-passivated lead sections formed from 13 fused benzene rings (middle), and the DOS peaks of the extended molecule section (right). DOS peak numbering matches the one appearing in Figs. S.11 and S.12. All energies are obtained in the absence of a magnetic field and are presented relative to the HOMO-LUMO gap center of the isolated* ***c-P10*** *molecule.*

S.9 The finite junction model

In the main text and Section S.8 above, we used a finite junction model (Fig. S.13) to help assign eigen-states found on the isolated ***c*-P10** ring to the DOS and transmittance probability peaks of a ***c*-P10** molecular ring coupled via acetylene-linkers to two polyacene leads. The finite junction model acts as a manageable intermediate between the isolated nanoring and the infinite molecular junction. For completeness, we include in Fig. S.14 the magnetic field dependence of the orbital energies of the finite junction model. The states that show strong magnetic field dependence are those which are delocalized across the junction, whereas the mid-gap states that are insensitive to the field are mainly localized on the lead sections. In Fig. S.15 the corresponding magnetic field dependence of representative MOs are presented for the isolated ring states and their finite junction model counterparts.

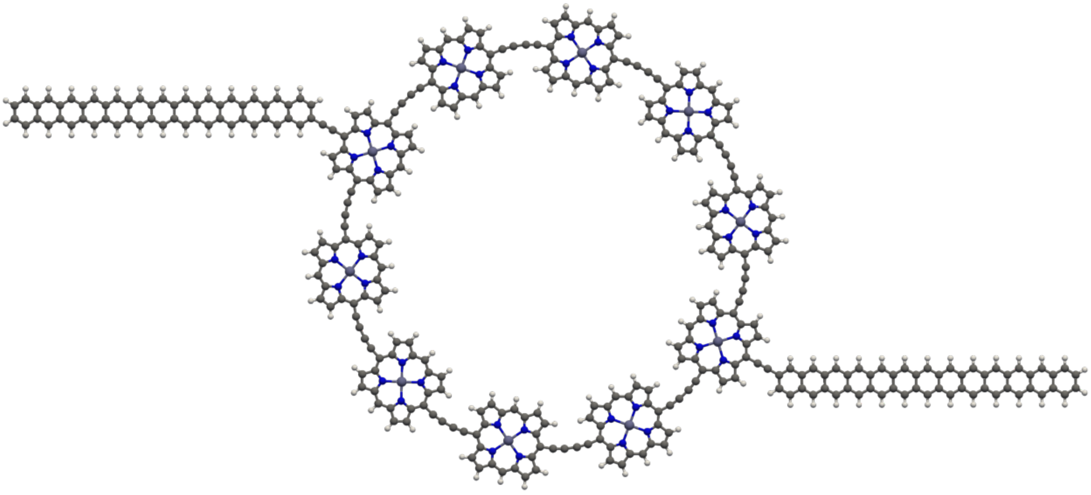

Fig. S.13. *The finite model junction created from a* ***c-P10*** *nanoring linked to polyacene chains formed from 13 fused rings via acetylene units.*

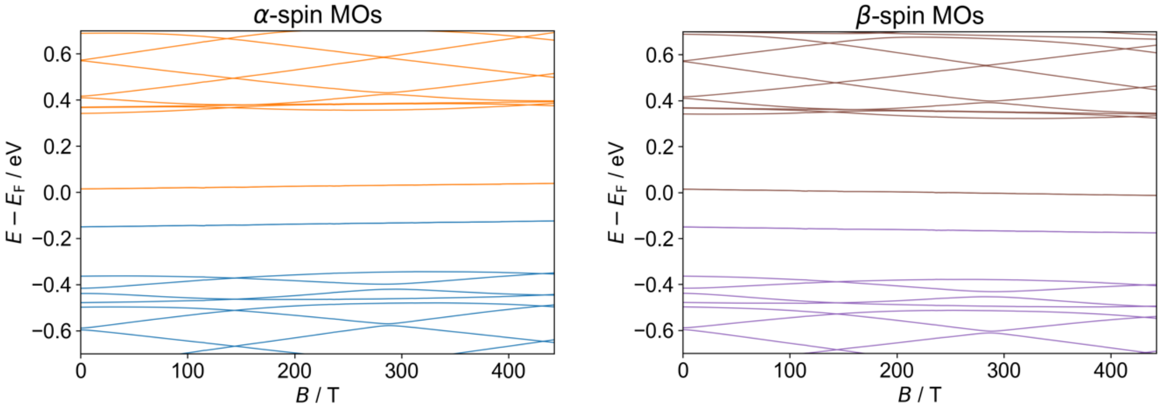

Fig. S.14. *GFN1-xTB-M1 α (left) and β (right) spin orbital energies of the finite junction model as a function of the perpendicular magnetic field strength. Orbital energies are given relative to the HOMO-LUMO gap center of the isolated* ***c-P10*** *nanoring in the absence of a magnetic field. Blue and orange (purple and brown) lines represent the occupied and unoccupied α-spin (β-spin) states, respectively.*

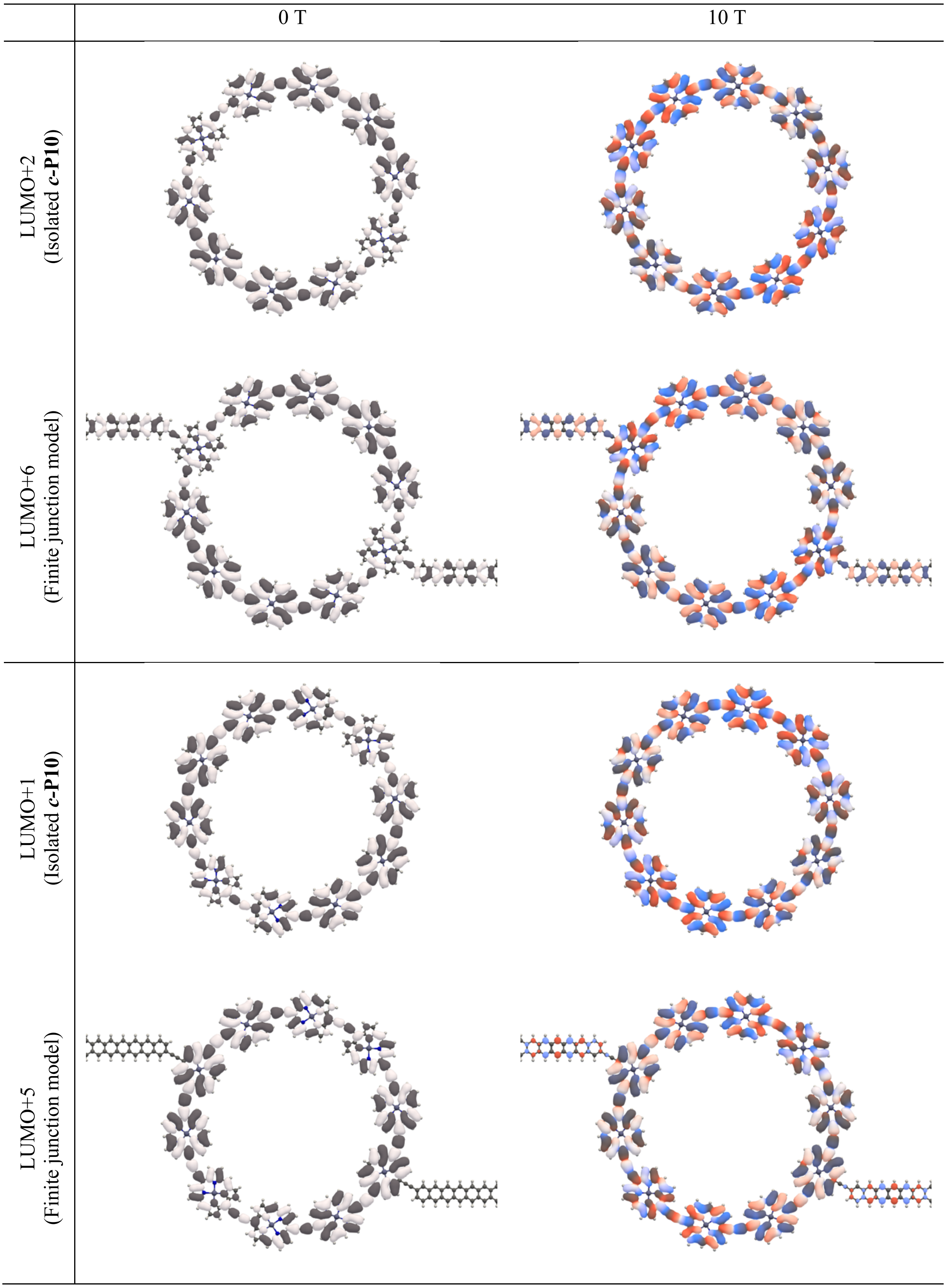

Fig. S.15. *The HOMO−4 to LUMO+2 molecular orbitals of the isolated* **c-P10** *ring (top) plotted in the absence of a magnetic field (left) and under a perpendicular magnetic field of 10 T (right) compared to their finite junction model counterparts (bottom). Orbital plots show isosurfaces with an isovalue of* $|\psi| = 0.0025\ a_0^{-3/2}$ *and are colored according to the phase of the orbital, where* $arg(\psi) \in (-\pi, \pi]$*, values are mapped to the color wheel in Fig. S.16.*

	0 T	10 T
LUMO (Isolated ***c*-P10**)	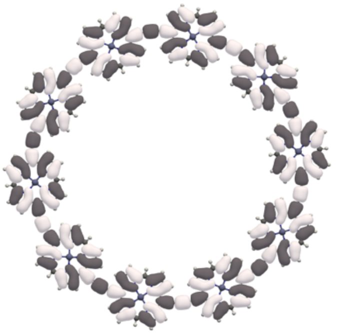	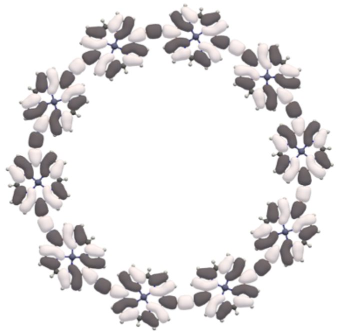
LUMO+2 (Finite junction model)	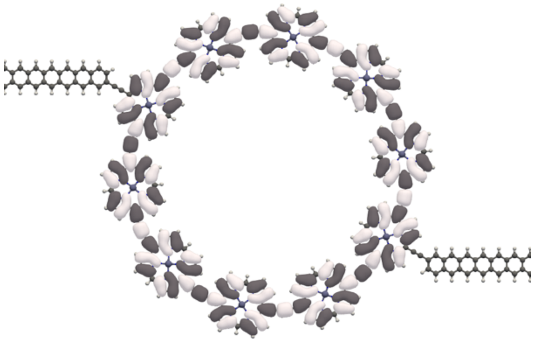	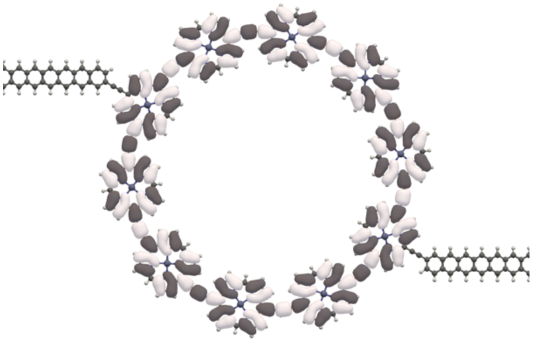
HOMO (Isolated ***c*-P10**)	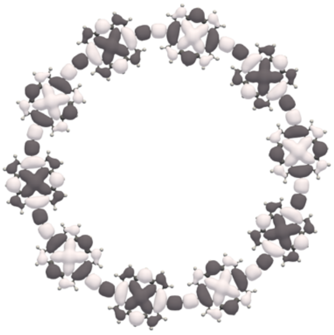	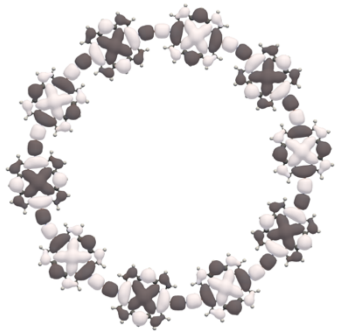
HOMO−2 (Finite junction model)	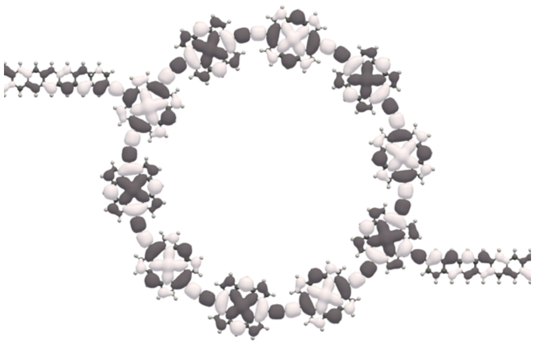	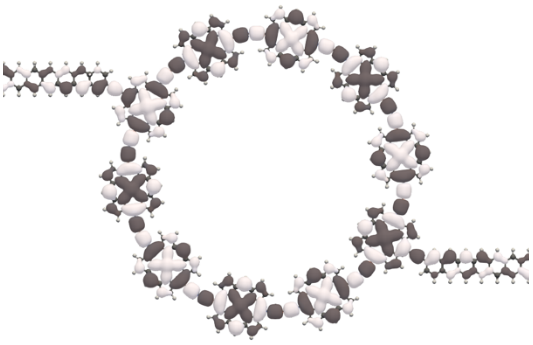

Fig. S.15. *continued*

	0 T	10 T
HOMO−1 (Isolated ***c*-P10**)	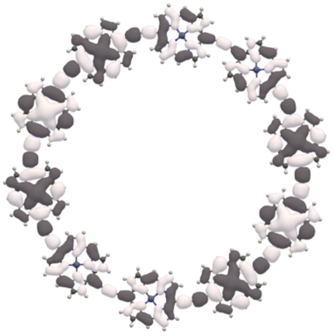	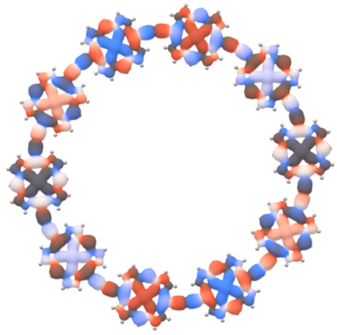
HOMO−3 (Finite junction model)	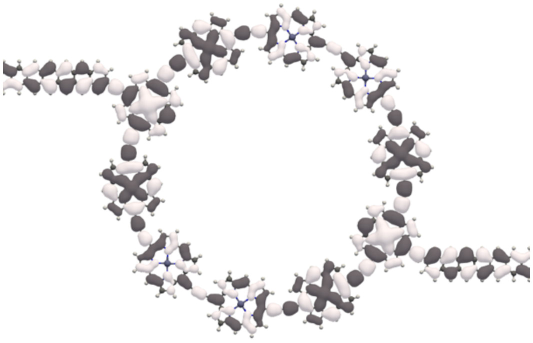	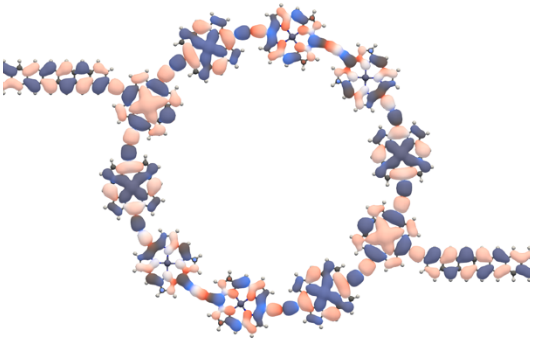
HOMO−2 (Isolated ***c*-P10**)	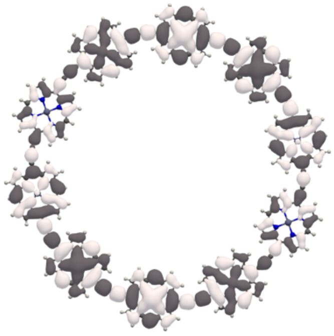	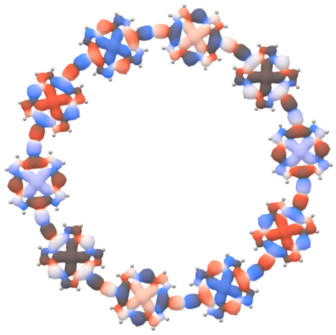
HOMO−4 (Finite junction model)	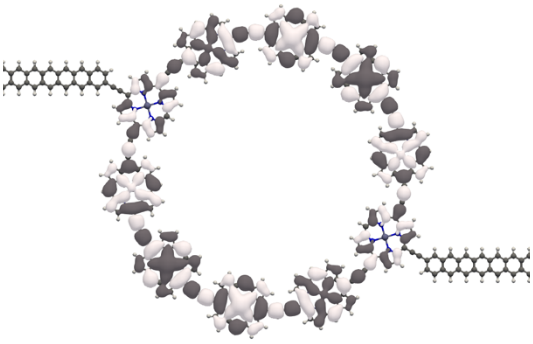	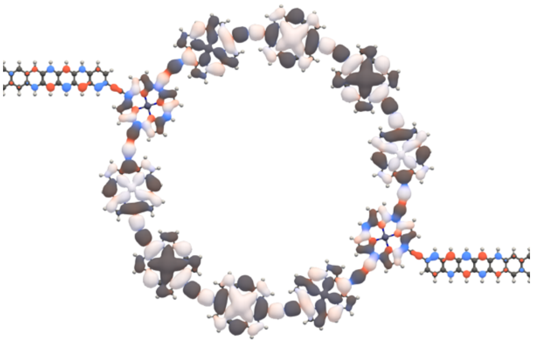

Fig. S.15. *continue*

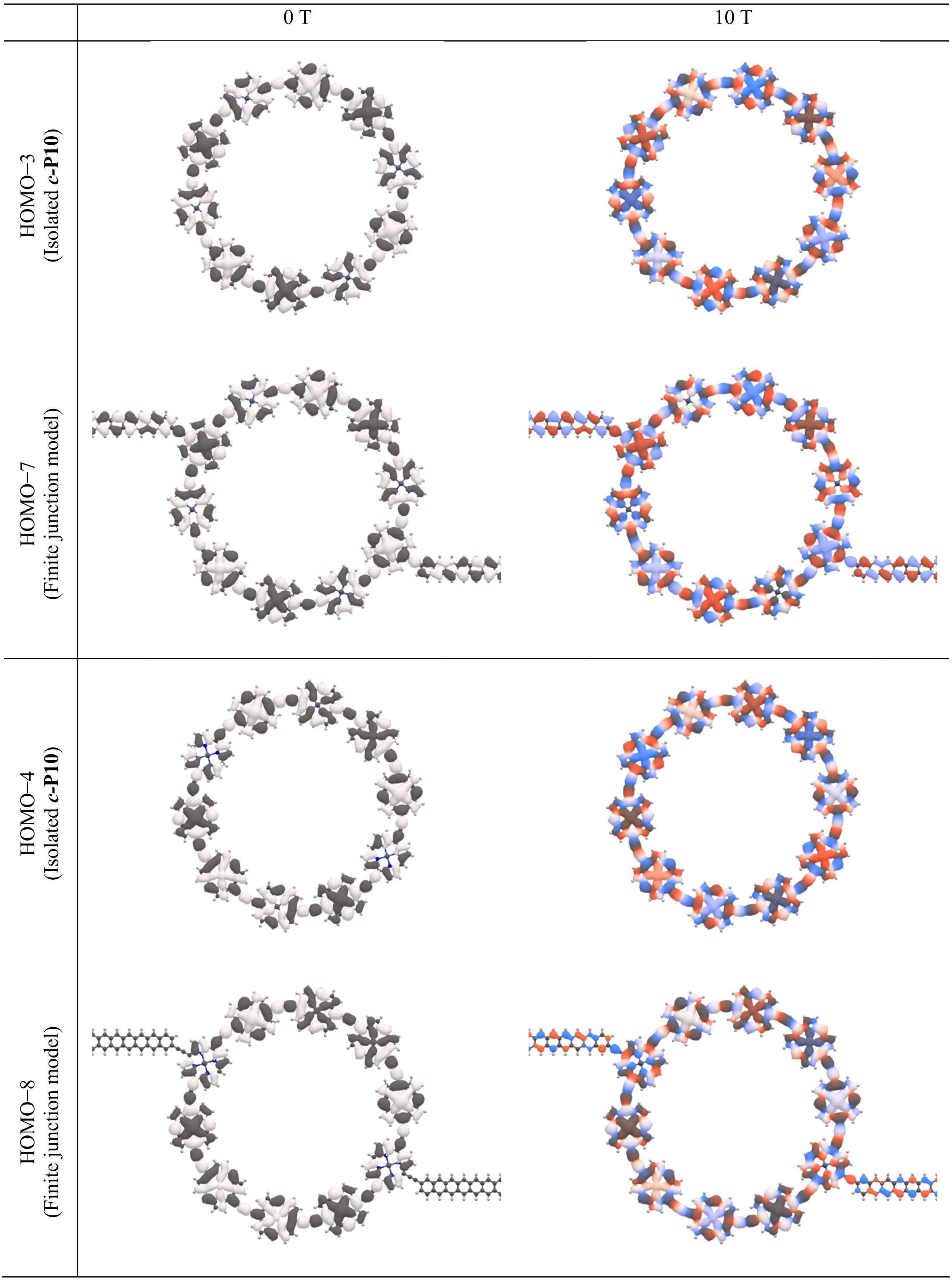

Fig. S.15. *continued*

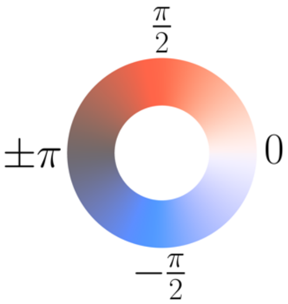

Fig. S.16. *The color wheel used to represent the phase of the molecular orbitals presented in Fig. S.15.*

S.10 Current switching at higher bias and electronic temperature

In the main article, we took advantage of the Fano resonances, seen in the transmission function, to modulate the current across the molecular interferometer. To maximize the effect, small 10 meV width Fermi transport windows with bath electronic temperatures of 0 K were used. Here, we present results with 50 meV windows that may be more easily achievable in an experimental setting and higher bath electronic temperatures, up to 50 K (see Fig. S.17). With the larger 50 meV windows, smaller current variations are obtained for a given magnetic field strength. For example, switching on a magnetic field of 10 T yields current variations of up to ~32–45% with the 10 meV transport window at 0 K (see main text Fig. 7), compared to variations of up to ~9–28% obtained with the 50 meV window at 0 K (Fig. S.17, upper row), ~5–13% at 25 K (Fig. S.17, middle row), and ~3–5% at 50 K (Fig. S.17, bottom row). Fig. S.18 presents the current change upon the application of a magnetic field of 10T as a function of temperature for a transport window of 50 meV, demonstrating that for the observation of AB interferometry in such porphyrin molecular rings, it is advisable to work at relatively low temperatures.

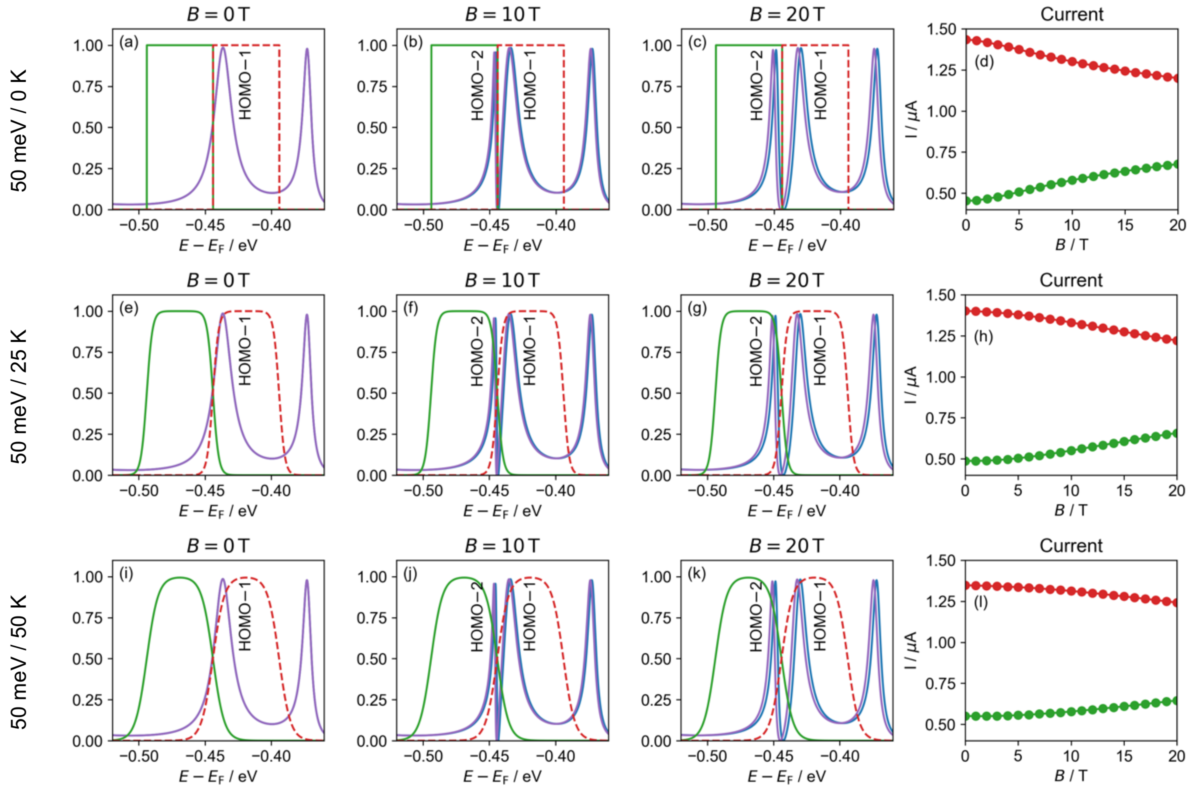

Fig. S.17. *(a–c,e–g,i–k) Transmission probability function for the α (blue) and β (purple) spin channels in the vicinity of the HOMO−1 and HOMO−2 (see Fig. 5 of the main text), calculated for a **c-P10** junction under perpendicular magnetic fields of (a,e,i) 0, (b,f,j) 10, and (c,g,k) 20 T. (d,h,l) Magnetic field dependence of the current, calculated using eq (6) of the main article with 50 meV wide Fermi windows (red and green) and electronic temperatures of 0 (top row), 25 (middle row), and 50 K (bottom row). Transmission probability peaks are labelled with the **c-P10** ring orbital they correspond to (see Section S.8).*

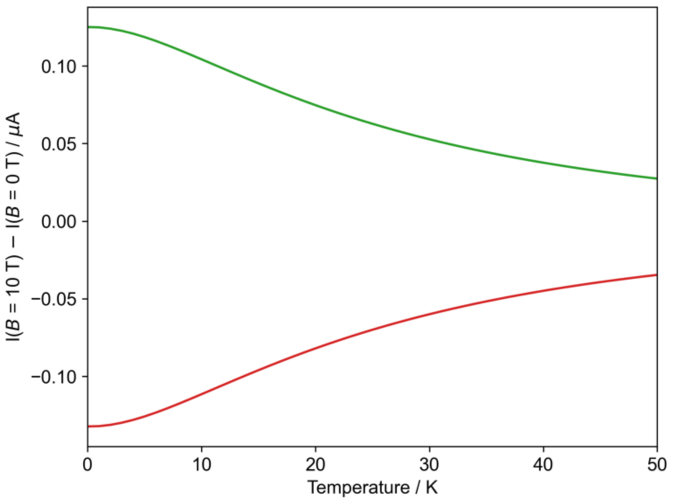

Fig. S.18. *The current change, after the application of a 10 T perpendicular magnetic field, as a function of temperature, calculated using 50 meV fermi transport windows gated at −469 (green) and −419 meV (red).*

S.11 Band structure calculations on infinite periodic linear ribbons

We carried out band structure calculations on edge-fused zinc and nickel coordinated porphyrin linear nanoribbons so as to compare their effective masses with the cyclic oligomers ***f*-P*N***. The geometries of these linear ribbons were optimized using the GFN1-xTB method within DFTB+ [9]. Periodicity was enforced along the *x*-axis with a Gamma centered 32×1×1 *k*-point mesh, and a vacuum region of 50 Å was applied along the *y* and *z*-axes to prevent spurious interactions between neighboring system images. The resulting band structures of the optimized geometries are shown in Fig. S.19. Effective masses were calculated for the lowest energy band above the Fermi energy at the Brillouin zone boundary resulting in electron effective masses of 0.0054 and 0.042 for the zinc and nickel coordinated porphyrin nanoribbons, respectively. Similar values (of opposite sign) for the hole effective masses were obtained for the highest energy band below the Fermi energy. Compared to PBE calculations for the nickel coordinated porphyrin nanoribbons [10, 11], we can see that GFN1-xTB underestimates effective masses so that we should expect the effective masses for the ***c*-P*N*** and ***f*-P*N*** nanoring to also be underestimated. The zinc coordinated porphyrin nanoribbon effective masses are smaller than those calculated for the ***f*-P*N*** series from the main article. These results follow expectations, given the decreasing effective masses with increasing ring size due to the decreased strain and increased coupling between porphyrin monomer units.

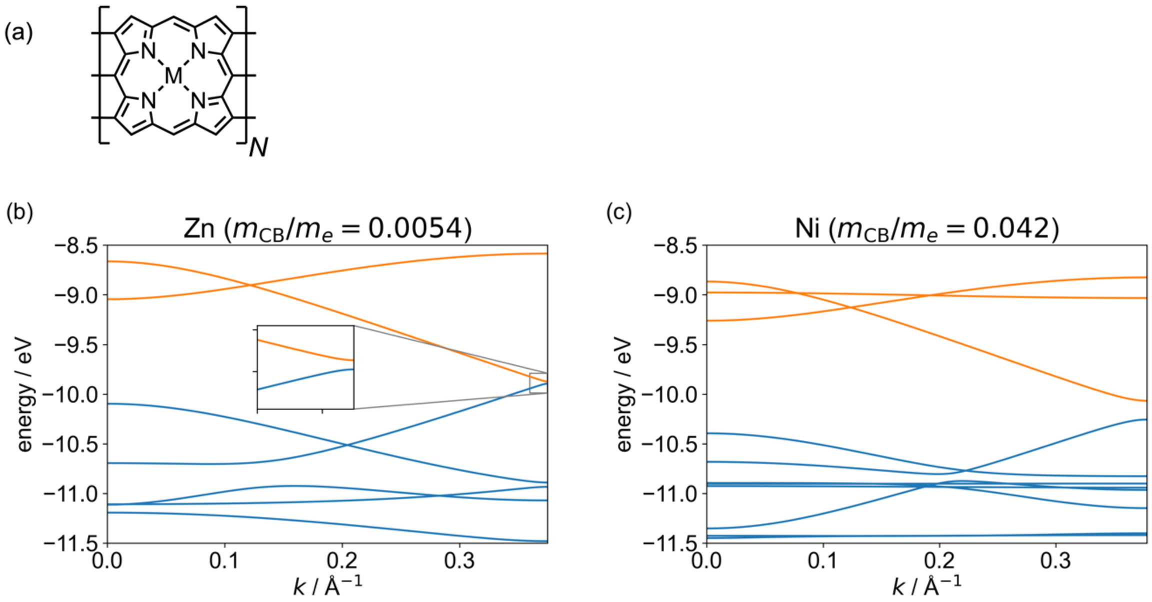

Fig. S.19. (a) Chemical structure of infinite periodic linear ribbons; (b) and (c) energy bands as a function of the wavevector k for M = Zn and M = Ni, respectively; occupied and unoccupied bands are shown by blue and orange lines, respectively. The band gaps are 0.022 eV (M = Zn) and 0.19 eV (M = Ni).

S.12 List of data files deposited on Zenodo

Data files relating to this manuscript are openly available via Zenodo; https://doi.org/10.5281/zenodo.13865479.

This data set includes:

./cpn

xyz files: Optimized geometries of the ***c*-P*N*** porphyrin nanorings using the GFN1-xTB method in the absence of a magnetic field.

txt files: Orbital energies of the ***c*-P*N*** porphyrin nanorings relative to its HOMO-LUMO gap center in the absence of a magnetic field calculated using the GFN1-xTB-M1 method where first number in each line is the magnetic field strength (Tesla) and the remaining numbers are the orbital energies (eV).

./cpn_flat

xyz files: Optimized geometries of the flattened ***c*-P*N*** porphyrin nanorings using the GFN1-xTB method in the absence of a magnetic field.

txt files: Orbital energies of the flattened ***c*-P*N*** porphyrin nanorings relative to its HOMO-LUMO gap center in the absence of a magnetic field calculated using the GFN1-xTB-M1 method where the first number in each line is the magnetic field strength (Tesla) and the remaining numbers are the orbital energies (eV).

./current

txt files: The calculated current as a function of the external magnetic field for the specified Fermi transport windows and electronic temperatures where the first number in each line is the magnetic field strength (Tesla) and the second number is the current (micro Amps).

./finite_junction

xyz files: Optimized geometries of the finite junction model using the GFN1-xTB method in the absence of a magnetic field.

txt files: Orbital energies of the finite junction model relative to the HOMO-LUMO gap center of the ***c*-P10** nanoring in the absence of a magnetic field calculated using the GFN1-xTB-M1 method where the first number in each line is the magnetic field strength (Tesla) and the remaining numbers are the orbital energies (eV).

./fpn

xyz files: Optimized geometries of the ***f*-P*N*** porphyrin nanobelts using the GFN1-xTB method in the absence of a magnetic field.

txt files: Orbital energies of the ***f*-P*N*** porphyrin nanobelts relative to its HOMO-LUMO gap center in the absence of a magnetic field calculated using the GFN1-xTB-M1 method where the first number in each line is the magnetic field strength (Tesla) and the remaining numbers are the orbital energies (eV).

./transmission

txt files: The transmission function calculation results using the NEGF+GFN1-xTB-M1 method for a number of field strengths and biased electron densities where the 1st, 2nd and 3rd numbers in each line are the energy (Hartree) relative to the HOMO-LUMO gap center of the ***c*-P10** nanoring in the absence of a magnetic field, transmission probability, and density of states.

./transmission_homo-1

txt files: The transmission function calculation results using the NEGF+GFN1-xTB-M1 method for energies around the HOMO-1 and HOMO-2 peaks for a number of field strengths where the 1st, 2nd and 3rd numbers in each line are the energy (Hartree) relative to the HOMO-LUMO gap center of the ***c*-P10** nanoring in the absence of a magnetic field, transmission probability, and density of states.

S.13 References

[1] S. Grimme, C. Bannwarth and P. Shushkov, *J. Chem. Theory Comput.*, 2017, **13**, 1989.

[2] C. Bannwarth, E. Caldeweyher, S. Ehlert A. Hansen, P. Pracht, J. Seibert, S. Spicher and S. Grimme*, WIREs Comput. Mol. Sci.*, 2021, **11**, e1493.

[3] C. Y. Cheng and A. M. Wibowo-Teale, *J. Chem. Theory Comput.*, 2023, **19**, 6226.

[4] F. London, *J. Phys. Radium.*, 1937, **8**, 397.

[5] R. D. Reynolds and T. Shiozaki, *Phys. Chem. Chem. Phys.*, 2015, **17**, 14280; A. Pausch and W. Klopper. *Mol. Phys.*, 2020, **118**, 21; T. J. P. Irons, G. David and A. M. Teale, *J. Chem. Theory Comput.*, 2021, **17**, 2166.

[6] G. L. Stoychev, A. A. Auer and F. Neese, *J. Chem. Theory Comput.*, 2017, **13**, 554.

[7] M. P. Lopez Sancho, J. M. Lopez Sancho and J. Rubio, *J. Phys. F: Met. Phys.*, 1984, **14**, 1205.

[8] M. P. Lopez Sancho, J. M. Lopez Sancho and J. Rubio, *J. Phys. F: Met. Phys.*, 1985, **15**, 851.

[9] B. Hourahine, B. Aradi, V. Blum, F. Bonafé, A. Buccheri, C. Camacho, C. Cevallos, M. Y. Deshaye, T. Dumitrică, A. Dominguez, S. Ehlert, M. Elstner, T. van der Heide, J. Hermann, S. Irle, J. J. Kranz, C. Köhler, T. Kowalczyk, T. Kubař, I. S. Lee, V. Lutsker, R. J. Maurer, S. K. Min, I. Mitchell, C. Negre, T. A. Niehaus, A. M. N. Niklasson, A. J. Page, A. Pecchia, G. Penazzi, M. P. Persson, J. Řezáč, C. G. Sánchez, M. Sternberg, M. Stöhr, F. Stuckenberg, A. Tkatchenko, V. W.-z. Yu and T. Frauenheim, *J. Chem. Phys.*, 2020, **152**, 124101.

[10] H. Zhu, Q. Chen, I. Rončević, K. E. Christensen and H. L. Anderson, *Angew. Chem. Int. Ed.,* 2023, **62**, e202307035.

[11] Q. Chen, A. Lodi, H. Zhang, A. Gee, H. I. Wang, F. Kong, M. Clarke, M. Edmondson, J. Hart, J. N. O'Shea, W. Stawski, J. Baugh, A. Narita, A. Saywell, M. Bonn , K. Müllen, L. Bogani and H. L. Anderson, *Nat. Chem.* 2004, **16**, 1133.
